# Enhancing smart home environments: a novel pattern recognition approach to ambient acoustic event detection and localization

**DOI:** 10.3389/fdata.2024.1419562

**Published:** 2025-01-23

**Authors:** Ahsan Shabbir, Abdul Haleem Butt, Taha Khan, Lorenzo Chiari, Ahmad Almadhor, Vincent Karovic

**Affiliations:** ^1^Department of Creative Technologies, Faculty of Computing and Artificial Intelligence, Air University, Islamabad, Pakistan; ^2^Research and Development Department, Detectivio AB, Göteborg, Sweden; ^3^Department of Electrical, Electronic and Information Engineering “Guglielmo Marconi, ” University of Bologna, Bologna, Italy; ^4^Department of Computer Engineering and Networks, College of Computer and Information Sciences, Jouf University, Sakaka, Saudi Arabia; ^5^Department of Information Management and Business Systems, Faculty of Management, Comenius University Bratislava, Bratislava, Slovakia

**Keywords:** ambient acoustic analysis, sound event detection, autonomous monitoring, machine learning, deep learning, ESPRIT, sound source localization

## Abstract

**Introduction:**

Ambient acoustic detection and localization play a vital role in identifying events and their origins from acoustic data. This study aimed to establish a comprehensive framework for classifying activities in home environments to detect emergency events and transmit emergency signals. Localization enhances the detection of the acoustic event's location, thereby improving the effectiveness of emergency services, situational awareness, and response times.

**Methods:**

Acoustic data were collected from a home environment using six strategically placed microphones in a bedroom, kitchen, restroom, and corridor. A total of 512 audio samples were recorded from 11 activities. Background noise was eliminated using a filtering technique. State-of-the-art features were extracted from the time domain, frequency domain, time frequency domain, and cepstral domain to develop efficient detection and localization frameworks. Random forest and linear discriminant analysis classifiers were employed for event detection, while the estimation signal parameters through rational-in-variance techniques (ESPRIT) algorithm was used for sound source localization.

**Results:**

The study achieved high detection accuracy, with random forest and linear discriminant analysis classifiers attaining 95% and 87%, respectively, for event detection. For sound source localization, the proposed framework demonstrated significant performance, with an error rate of 3.61, a mean squared error (MSE) of 14.98, and a root mean squared error (RMSE) of 3.87.

**Discussion:**

The integration of detection and localization models facilitated the identification of emergency activities and the transmission of notifications via electronic mail. The results highlight the potential of the proposed methodology to develop a real-time emergency alert system for domestic environments.

## 1 Introduction

Smart home technology has transformed our living environments into more secure, convenient, and comfortable spaces. By integrating advanced monitoring and automation systems, these technologies detect and respond to external stimuli, particularly auditory signals. Through acoustic event detection and sound localization, smart homes can autonomously monitor activities, identify specific events, and pinpoint sound sources. This functionality enhances security by detecting potential threats, such as intrusions or fires, while also improving convenience by recognizing doorbells, alarms, and voice commands. Furthermore, by mapping and interpreting the acoustic environment, smart homes can dynamically adjust their responses to meet the unique needs of their occupants (Ni et al., 2015).

Detection of sound events in dynamic environments poses considerable challenges due to the presence of background noise from sources such as music, conversations, and electrical appliances, which can obscure target auditory signals (Mesaros et al., 2021). To improve the clarity of auditory signals, Pre-processing techniques are employed, including filtering out extraneous noise and segmenting the signal to ensure adequate representation of all event types.

Recent studies have leveraged machine learning methods to analyze infrequent yet critical events, such as fire alarms and security breaches (Yang et al., 2022; Kim and Jung, 2023). These investigations utilize a range of acoustic features derived from time, frequency, and cepstral analysis to train machine learning models, enabling the identification of characteristics associated with critical sound events. Notably, research by Mesaros et al. (2021), highlighted the efficacy of features such as mel-frequency cepstral coefficients in effectively capturing the essential properties of various sound events (Ni et al., 2015).

While much of acoustic research has focused on event detection, the ability to accurately locate the source of sound events within a home is equally important. Identifying the location of a sound, such as the room where a fire alarm is sounding or where glass has broken, provides valuable information for both the system and users. Sound source localization methods, like ESPRIT, use time difference of arrival measurements from multiple microphones placed throughout the home to determine the sound's origin (Jiang et al., 2024; Wang et al., 2024).

Sound localization faces significant challenges in complex environments where echoes, reverberation, and multiple simultaneous sound sources are present. Traditional methods like delay-and-sum beamforming and MUSIC (Rascon and Meza, 2017) have been used, but their performance deteriorates in highly reverberant or noisy environments. To improve the accuracy in dynamic home environments, modern approaches integrate these classical techniques with machine learning models.

Despite advancements in acoustic event detection and localization, key challenges remain. Most systems focus on either detection or localization, lacking a unified framework that can handle both in real time. As Ni et al. (2015) pointed out, designing algorithms to handle the dynamic nature of sound events in changing home environments affected by furniture arrangements, new devices, or occupancy variations is still a challenge. Additionally, false-positives and -negatives are common in noisy environments, where non-emergency sounds may be misclassified as emergencies or true events may be missed due to overlapping noise. Enhancing feature selection and classification models, using techniques like recursive feature elimination and SHAP, can improve accuracy and model explanation to address these issues. Moreover, there is a lack of systems that integrate these capabilities with automated alert mechanisms, such as sending notifications of an emergency event to promptly notify relevant parties about emergencies (Al-khafajiy et al., 2019). As highlighted in Stowell et al. (2018), designing algorithms capable of managing the dynamic nature of sound events in different home contexts remains a challenge.

Existing research predominantly concentrates on either acoustic event detection or sound source localization within smart home environments, but rarely integrates both in a unified framework, particularly for emergency activities. For instance, while Valenzise et al. (2007) developed a system for shriek and gunfire detection and localization in audio-surveillance contexts, their approach does not extend to smart home environments or include automated alerting mechanisms via electronic mail. Similarly, Dennis et al. (2013) combined spectral and spatial features for sound event detection but did not integrate localization with emergency alert systems.

We propose a system that addresses the limitations of current smart home technologies by integrating state-of-the-art deep learning for event detection together with advanced localization algorithms, offering a real-time solution that adapts to complex acoustic environments. This system combines multiple feature extraction methods and machine learning models for a precise identification of acoustic events, followed by the ESPRIT algorithm for sound source localization. A real-time audio input module continuously monitors the environment, analyzing sounds and triggering emergency alerts when necessary. By merging deep learning-based detection with robust localization, this framework can enhance the accuracy and responsiveness of smart home systems, improving both safety and usability.

To stress the current status of home acoustic automation, Wilhelm and Wahl (2024) highlighted the need for integrated systems that combine detection and localization for emergency response in smart homes but noted that such systems are still underdeveloped.

## 2 Literature review

The detection and localization of acoustic activities in home environments have seen significant advancements, driven by applications in assisted living, home automation, and safety monitoring. This review examines key developments, highlighting foundational techniques, advancements in machine learning and deep learning, challenges in complex environments, and the integration of detection and localization systems. A critical evaluation of the existing studies reveals limitations and gaps that our research aims to address.

### 2.1 Foundational techniques in acoustic localization

Early research established fundamental principles for sound source localization (SSL) in complex acoustic environments. Middlebrooks and Green (1991) pioneered the use of temporal regularities to enhance SSL by analyzing the temporal structure of sound sources. By leveraging timing differences in sound wave arrivals, they improved localization performance. However, their approach was limited in handling the diversity and overlapping acoustic events typical in real-world home environment, where multiple sound sources and reflections complicate accurate localization.

Building on these foundational methods, Kameoka et al. (2015) addressed the challenges in urban acoustic activity detection, emphasizing the need for precise sound differentiation in noisy environments. Their robust classification models handled diverse audio signals but were tailored to urban contexts, differing from indoor environments in sound sources and acoustic properties. While their work advanced SSL techniques, it did not fully account for the unique challenges posed by indoor reverberations and the variability of home soundscapes.

### 2.2 Advancements in machine learning and deep learning for acoustic classification

The emergence of machine learning techniques introduced new possibilities for acoustic classification and localization. DeVore et al. (2017) applied particle swarm optimization to enhance support vector machine (SVM) classifiers for recognizing specific acoustic signals. This optimization improved classification accuracy, highlighting the importance of feature extraction and algorithm tuning. However, SVMs may struggle with large-scale data and complex nonlinear relationships inherent in acoustic signals, limiting scalability in dynamic home environments.

The advent of deep learning marked a significant shift. Hyun et al. (2016) introduced a hybrid model combining long short-term memory (LSTM) networks and convolutional neural networks (CNNs) to capture both temporal and spatial features of indoor acoustic activities. Diraco et al. (2019) extended this approach to monitor changes in older individuals' daily routines using one-class SVMs and convolutional autoencoders, achieving 88% accuracy in detecting deviations. While these models demonstrated effectiveness, they often lacked interpretability and required extensive labeled data, posing challenges for deployment in sensitive environments like smart homes.

Further advancements included methods leveraging spatial data to improve classification accuracy. Basbug and Sert (2019) employed spatial pyramid pooling in CNNs, enhancing performance but increasing computational demands unsuitable for resource-constrained devices. Mushtaq and Su (2020) proposed an ensemble of CNNs with data augmentation techniques, achieving high accuracy rates. However, the computational complexity and the need for large datasets limited their practicality for real-time applications in home environments.

### 2.3 Challenges in complex acoustic environments

Differentiating sounds in noisy and reverberant environments remains a significant challenge. Ciaburro and Iannace (2020) emphasized the difficulty of acoustic activity detection in urban environment, proposing models tailored to complex noise profiles. Nzimbakani et al. (2020) developed an SSL method integrating particle filtering and time–frequency analysis to reduce false localization in noisy home environments. While effective, these approaches involved sophisticated signal processing techniques and high computational costs, hindering real-time application.

Handling overlapping acoustic events is another notable deficiency. Many systems struggle to differentiate concurrent sounds or accurately localize sources in reverberant conditions, reducing effectiveness in real-world scenarios. Bonet-Solà and Alsina-Pagès (2021) concluded that no single feature extraction method is universally applicable, emphasizing the need for adaptable systems that can handle varying acoustic conditions.

### 2.4 Integration of detection and localization for emergency events

Despite advancements, there is a lack of integrated frameworks combining acoustic event detection and localization for emergency activities in home environments. Min et al. (2018) developed an emergency sound detection system using deep learning but omitted localization capabilities. Zhang et al. (2024) presented a real-time detection and localization system without focusing on emergency activities or electronic alert communication.

Some studies have begun integrating detection and localization to enhance smart home responsiveness. Dennis et al. (2013) combined spectral and spatial features for improved localization accuracy in real-life recordings. Valenzise et al. (2007) developed a system for scream and gunshot detection and localization in audio surveillance, highlighting the importance of such integration for emergency events. However, these systems were designed for surveillance contexts and may not directly apply to home environments due to differing acoustic properties and privacy concerns.

Thakur and Han (2021) focused on indoor localization to accurately detect a person's position, aiming to expedite medical assistance during emergencies. Using a big-data-driven approach and machine learning techniques like Random Forests, they achieved an accuracy of 81.36%. While their method improved localization, it did not integrate acoustic event detection or automated alerting mechanisms essential for comprehensive emergency response.

### 2.5 Limitations and gaps in current studies

A critical evaluation of existing studies reveals several limitations:

- **Lack of integrated systems**: Most studies focuses on either detection or localization, rarely combining both for emergency activities. This separation limits the effectiveness of smart home systems in providing timely assistance.
- **Handling overlapping events**: Systems struggle with concurrent sounds and reverberant conditions, reducing accuracy and reliability in real-world home environments. The inability to manage overlapping acoustic events limits the applicability of these systems in dynamic home environments.
- **Lack of automated alerts**: Few systems incorporate mechanisms to convey notifications via electronic means like email or SMS. The absence of automated alerting diminishes practical utility during emergencies when immediate communication is essential.
- **Adaptability and scalability**: Many models focus on specific activities or environments without adapting to the dynamic nature of real homes. The lack of scalability and adaptability hinders long-term applicability, as home environments and occupant behaviors change over time.

Despite significant advancements, there remains a critical research gap in developing comprehensive frameworks that integrate acoustic event detection and localization for emergency activities in smart homes, coupled with automated alert mechanisms. Addressing these deficiencies is essential for enhancing the practical applicability and reliability of acoustic monitoring systems in real-world environments.

Our research aims to fill this limitation by proposing a system that not only detects and localizes emergency acoustic events but also transmits immediate notifications through electronic communication channels. By integrating advanced deep learning techniques for accurate detection with efficient localization algorithms and an automated alert system, we address the critical need for timely assistance during emergencies. This approach enhances responsiveness and safety, particularly for people in need such as the older people or individuals with disabilities.

## 3 Research methodology

**Figure 1:**
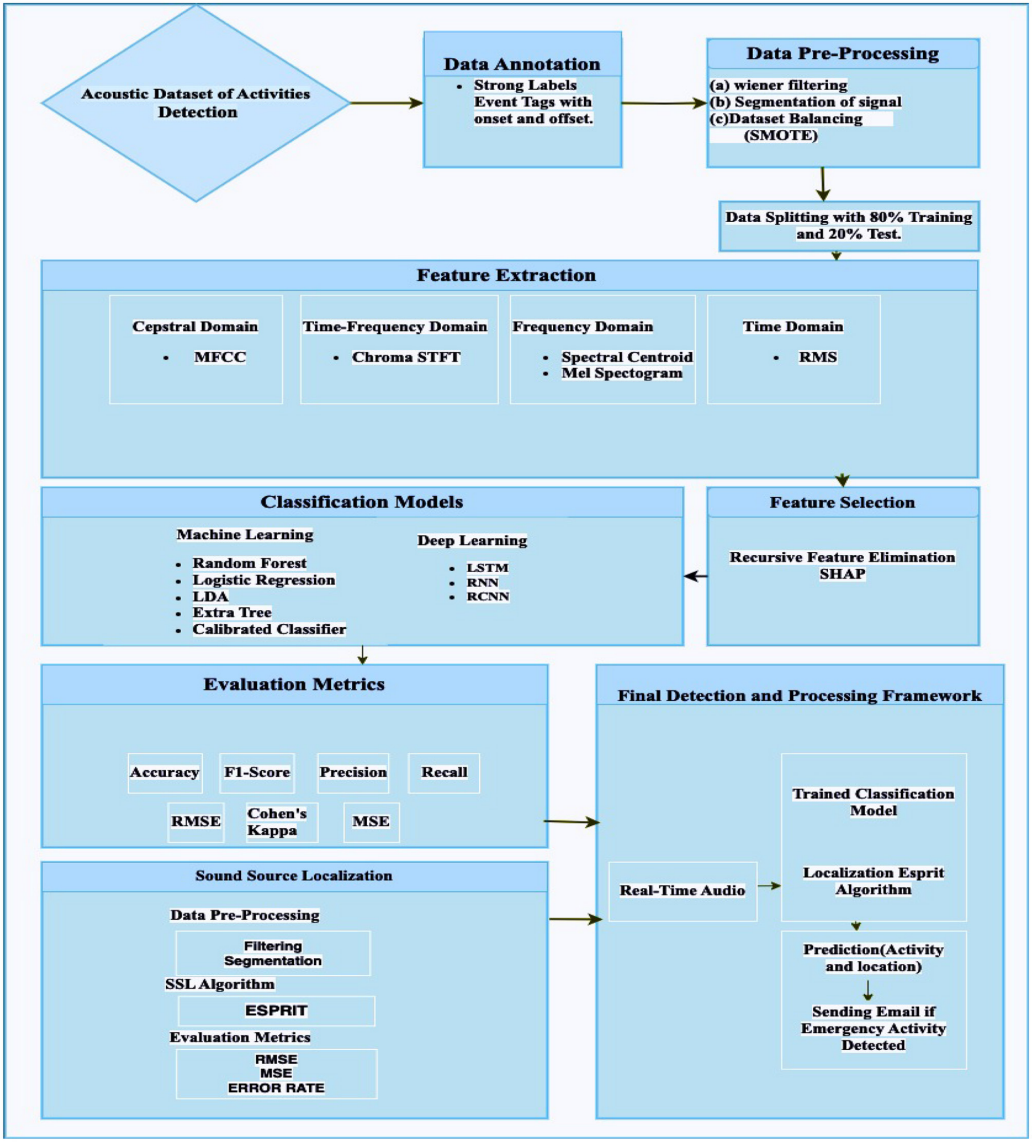
Flowchart of the ambient acoustic event detection.

The methodology of the study for activity detection and localization, as well as the sending of an emergency signal, is depicted in the Figure 1.

- **Data acquisition and annotation**: The study starts with the acquisition and annotation of an acoustic dataset with strong labels marking event onset and offset.
- **Data pre-processing**: The quality of the data is enhanced by the application of pre-processing stages, signal segmentation, noise reduction, and dataset balancing.
- **Feature extraction**: The audio signals are subsequently effectively defined by extracting features from a variety of domains.
- **Feature selection and development of model**: Feature selection techniques are used to improve performance, and machine learning and deep learning models are used for even detection.
- **Sound source localization**: The pipeline was developed for sound localization where the ESPIRT model was applied for the localization of the sounds.
- **Integration of system**: The framework integrates a real-time emergency detection system for deployment in diverse acoustic environments and incorporates sound source localization with the conventional localization algorithm ESPRIT to identify activity sources.

In the next section, a more detailed explanation of the methodology outlined above is provided. The current study adopts a method based on a literature review and addresses the limitations identified in our previous study (Lundström et al., 2016). The focus is primarily on developing data collection protocols, as well as assessing and validating the recorded acoustic data. This study provided a comprehensive pipeline for the classification and localization of daily living activities.

### 3.1 Data collection

**Figure 2:**
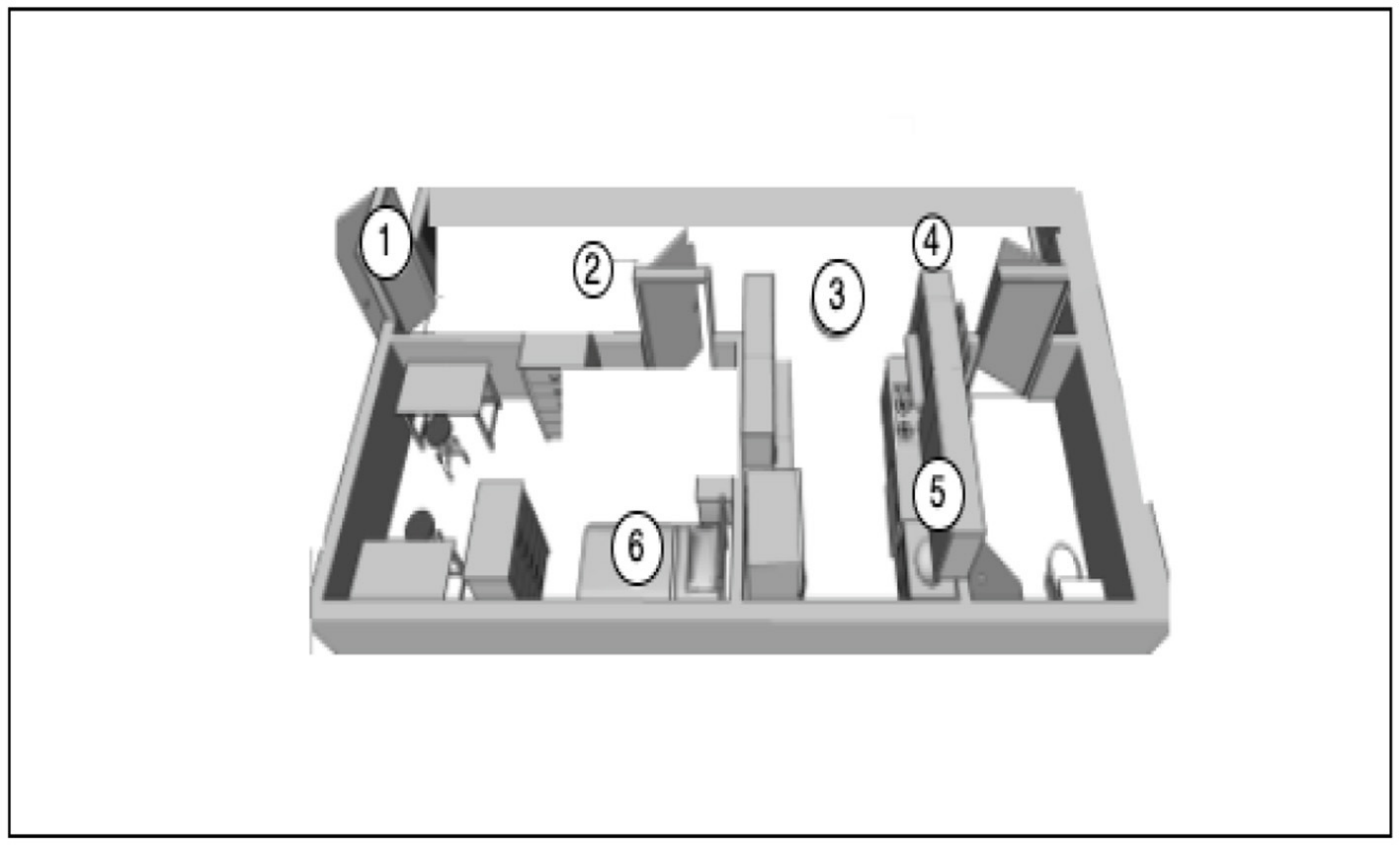
The floor plan of HINT divided into multiple areas. Labels in the figure indicate capabilities.

Preliminary studies and data collection were done in Halmstad Intelligent Home (HINT). This represents a controlled environment suitable for performing initial experiments. A series of data collection experiments were performed in the home environment, where audio data was collected from actual in-use apartments (hired to tenants by HFAB). This data was used to build signal processing and machine learning models. HINT has been equipped with more than 60 sensors, including one “smart home in a box” kit [1] to detect the current state of the environment and its occupants. Magnetic switches detect the opening/closing of doors (label 1 in Figure 2). Contact/touch sensors are positioned in the sofa and under the seat cushion to detect occupancy (label 2 in Figure 2). Passive infrared (PIR) sensors are positioned to detect motion or occupancy in the different areas (label 3 in Figure 2). Magnetic switches detect the opening/closing of cabinet's doors and drawers (label 5 in Figure 2). Load-cells integrated into the bed frame measure weight and bed entrances and exits, and pressure sensitive sensors under the mattress detect vital signs (label 6 in Figure 2). Motor actuators in the adjustable bed enable different bed positions to be selected (label 6 in Figure 2). A vacuum cleaner-like robot (label 4 in Figure 2) can navigate autonomously in the apartment and respond to detected anomalies, such as a fall.

In addition to microphones, and to enhance privacy, thermal cameras were also used for data annotation purposes. The initial experimental set-up in the intelligent home laboratory shows that two care phones as well as a separate four-microphone setup were deployed. The four-microphone set-up utilizes four similar microphones to those used in the Carephone, which will make comparisons more valid. The reason for having two Carephones is that they are positioned in different rooms. This will allow us to explore whether a single care phone (probably in the living room) can sense what is happening in another room.

In this study, all the activities have been recorded from one subject in order to standardized the protocol and data analysis as given below.

The protocols were as follows:

- Person enters the home.
- Goes to the bedroom and has some rest.
- Goes to the bathroom.
- Goes to the kitchen and open the cabinet.
- Again goes to the bedroom.

**Figure 3:**
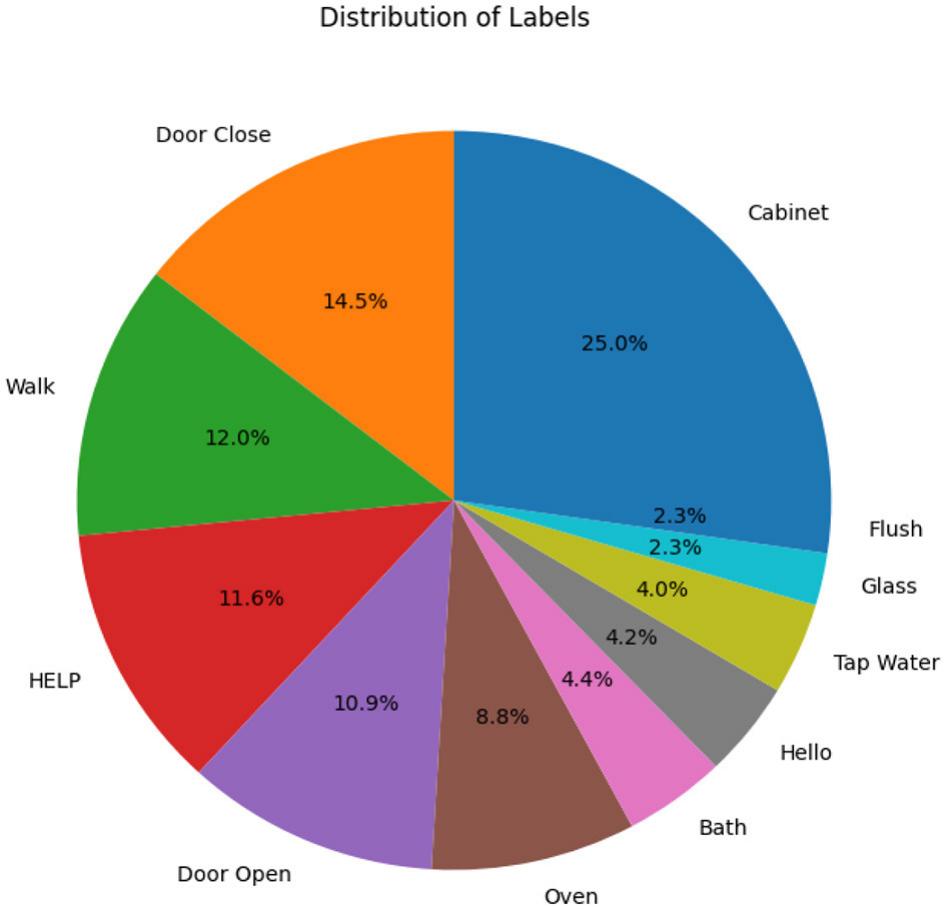
Distribution of different activities across the acoustic data set.

The home environment shown in Figure 2 consists of a bedroom, a corridor, a kitchen, and a bathroom. Six microphone sensors collected the activities in the home from one subject for 100 min. The data set size is 100 min with activities randomly placed from 11 classes mentioned in Figure 3, recording having a sampling rate of 44,000 Hz.

**Figure 4:**
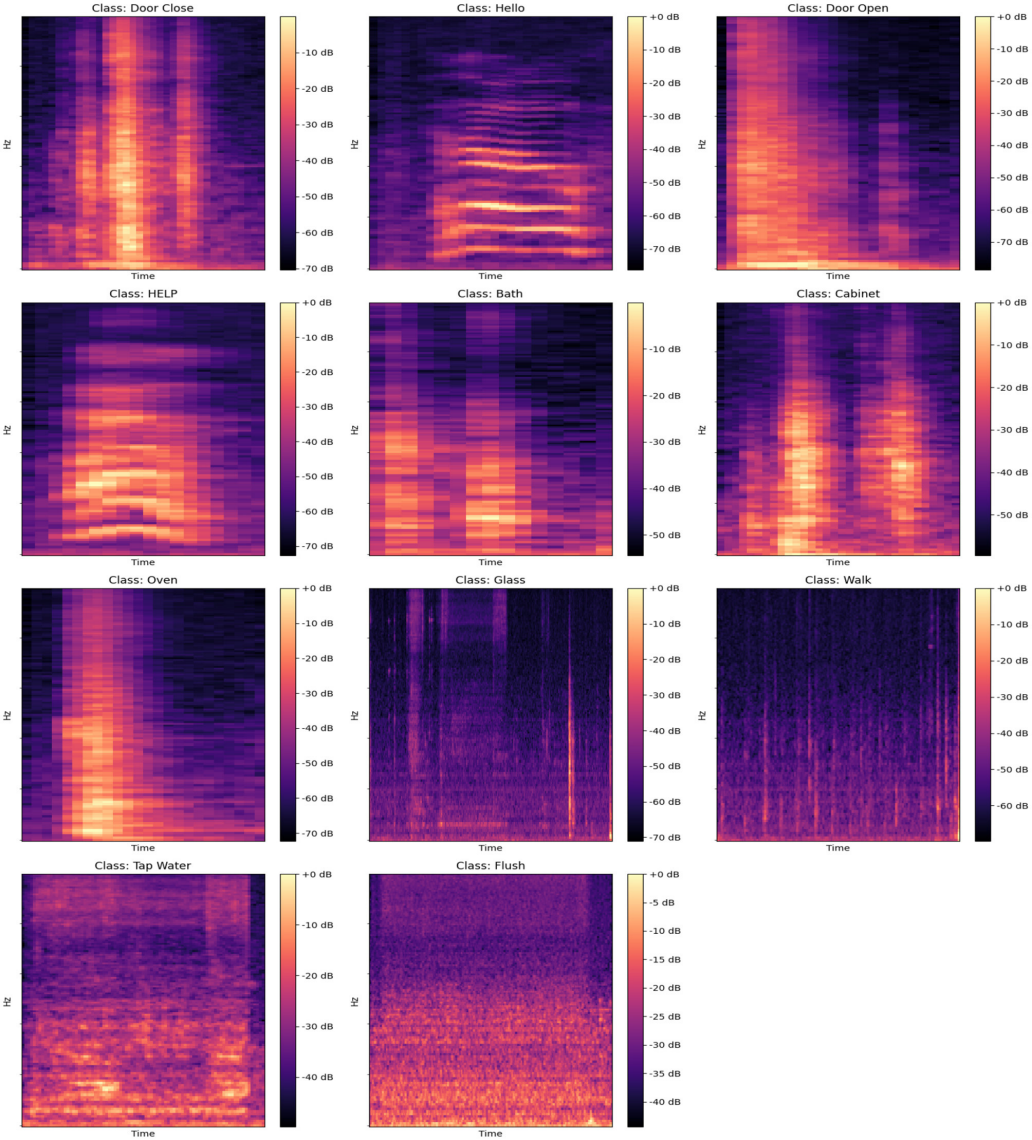
Spectrograms for different acoustic activities, including “Door Close,” “Hello,” “Door Open,” “HELP,” “Bath,” “Cabinet,” “Oven,” “Glass,” “Walk,” “Tap Water,” and “Flush,” showcasing the frequency and amplitude patterns over time for each class.

Figure 4 presents the spectral representations of acoustic activities for 11 distinct classes: “Door Close,” “Hello,” “Door Open,” “Help,” “Bath,” “Cabinet,” “Oven,” “Glass,” “Walk,” “Tap Water,” and “Flush.” Each spectrogram provides a comprehensive view of the time–frequency characteristics unique to these activities. The spectrograms reveal prominent peaks, corresponding to moments of high-energy acoustic activity, with signal intensities reaching up to 0 dB and significant frequencies spanning from approximately 0 Hz up to 4,096 Hz. These peaks highlight essential temporal and spectral features that are critical for distinguishing and classifying specific activities.

Lower intensity regions, typically observed within the range of –50 to –70 dB, represent periods of reduced acoustic activity or background noise. Activities such as “Door Close” and “Cabinet” exhibit distinct, high-energy bursts at frequencies between 500 and 2,000 Hz, indicating characteristic sound events. In contrast, continuous activities such as “Tap Water” and “Flush” show energy distributed more diffusely across a broader range, with prominent components below 1,000 Hz. The “Hello” and “Help” classes, associated with speech, display complex patterns with significant energy up to 3,000 Hz, while the “Glass” and “Walk” classes show more scattered energy across the entire range, often extending beyond 3,000 Hz.

The spectrograms emphasize how the distribution of energy varies across different activities. For example, “Oven” and “Door Open” display more isolated, periodic high-energy sections between 1,000 and 2,500 Hz, whereas “Walk” and “Flush” include widespread, lower-energy components spanning the entire frequency band up to 4,096 Hz. These detailed representations enable the identification of unique acoustic signatures associated with each activity.

While these spectrograms inherently capture both signal and noise, the subsequent sections will briefly outline the application of de-noising techniques, such as Wiener filtering, to enhance the signal-to-noise ratio. This approach aims to optimize feature extraction and improve the clarity of acoustic data, thereby facilitating more effective analysis in diverse real-world environments. This visual and frequency-based examination underscores the importance of recognizing activity-specific acoustic patterns for robust activity detection.

#### 3.1.1 Annotation

Annotations were labeled by human experts who listened to the audio data and identified the activity signals, determining their starting and ending points. The metadata is shown in Table 1, showing the onset starting point of the signal and offset ending point of the signal concerning the class name.

**Table 1:** Details of collected acoustic activities including file name, onset, offset, and activity label.

**File name**	**Onset (seconds)**	**Offset (seconds)**	**Label**
Final Track.wav	19.037045	20.188752	Door close
Final Track.wav	22.221177	23.169641	Hello
Final Track.wav	23.305136	24.050359	Door open
Final Track.wav	40.783989	41.359842	HELP
Final Track.wav	42.477676	43.087403	HELP

#### 3.1.2 Noise removal

**Figure 5:**
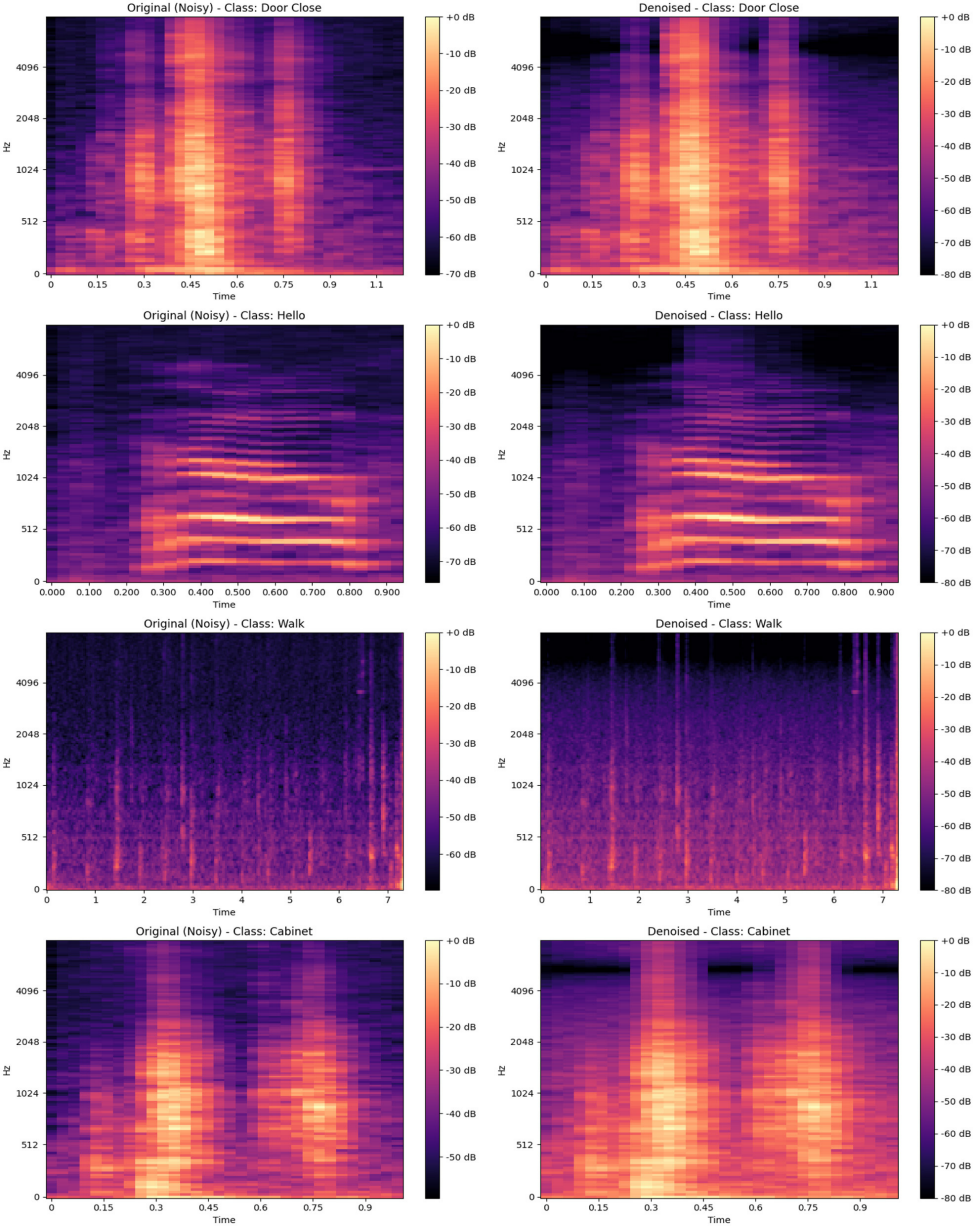
Comparison of original (noisy) and denoised spectrograms for various classes: “Door Close,” “Hello,” “Walk,” and “Cabinet,” illustrating the effectiveness of noise reduction in enhancing acoustic signal clarity.

Wiener filtering is a statistical method that is frequently employed in acoustic signals to reduce noise while preserving its important signal characteristics (Bentler, 2005). This is because it can reduce the mean square error (MSE) between the original and denoised signals. This adaptive filter can estimate the power spectral densities of each signal and noise while selectively reducing noise while preserving essential signal components, as it operates on the assumption that both signal and noise exhibit stable statistical characteristics over time. Wiener filtering dynamically adjusts to noise and signal characteristics, balancing noise suppression with signal fidelity, in contrast to alternative methods such as spectral subtraction, which can introduce median filtering, which struggles with frequency-specific noise, and wavelet thresholding, which may lead to distortion. This characteristic is particularly advantageous in audio processing, as demonstrated by our comparative spectrogram analysis. Figure 5 displays the spectrograms of four acoustic activity samples: “Door Close,” “Hello,” “Walk,” and “Cabinet,” with each activity shown in its original (noisy) form alongside its denoised counterpart processed using Wiener filtering. The spectrograms illustrate the significant differences in frequency distribution and intensity across these activities, with peaks reaching up to 0 dB in energy-rich segments and noise levels typically ranging between –50 and –60 dB. The application of Wiener filtering markedly enhances signal clarity, reducing background noise to below –70 dB while preserving the structural integrity of the original sound. This enhancement facilitates a clearer distinction of key acoustic features, essential for accurate analysis and classification. Such denoising proves effective for high-quality signal processing, striking a balance between signal preservation and noise reduction, thus solidifying its role as a reliable method for acoustic data analysis in complex environments.

#### 3.1.3 Data splitting

To construct the model with an 80% to 20% ratio, the data was divided into training and testing sets and subjected to 10-fold cross-validation. It is crucial to investigate the issue of imbalanced data, which occurs when the distribution of classes in the dataset is highly skewed or unequal, resulting in a majority class and one or more minority classes (Patel et al., 2020). In the context of sound activity detection and localization, imbalanced data can lead to biased algorithms that favor the majority class, ultimately resulting in poor performance for minority classes. The issue of unequal data can be addressed through the implementation of various approaches, including the over-sampling of the minority class, the under-sampling of the majority class, or a combination of both (Haque et al., 2014). One prominent over-sampling strategy employed in this study is the synthetic minority over-sampling technique (SMOTE), which creates synthetic samples for the minority class by interpolating between extant minority samples (Haque et al., 2014). Compared to its alternatives, such as straightforward random over-sampling and under-sampling, SMOTE offers significant advantages in scenarios involving imbalanced datasets. While random over-sampling merely duplicates existing minority class samples, leading to over-fitting and a lack of new information, SMOTE enriches the feature space by generating diverse, synthetic data points. Conversely, under-sampling the majority class risks eliminating valuable information and could worsen the imbalance. By leveraging SMOTE, we not only enhance the representation of minority classes but also provide the model with a more informative training set that reflects the complexities of the data, thereby enhancing performance across all classes.

### 3.2 Feature extraction

#### 3.2.1 Cepstral domain features

Cepstral domain features are features derived from a signal's cepstral representation. They are widely used in machine learning and signal processing applications, especially audio and voice analysis as mentioned in the one of recent studies (Sharma et al., 2020). The inverse Fourier transform of the signal's spectrum logarithm yields the cepstrum. It is a measurement of the rate of change in various spectrum bands. The cepstral domain is widely used in speech and audio processing because it is particularly helpful in isolating the source and filter properties of signals. For example, MFCCs. It is the feature we extracted in our study from the cepstral domain.

##### 3.2.1.1 MFCC

To capture the phonetically significant aspects of speech, filters are linearly spaced at low frequencies, and at the point where frequencies are high, logarithmically utilized. The critical bandwidths of the human ear fluctuate with frequency, which is the basis for MFCC features. The mel-frequency cepstrum, which is close to the response of the voicing system of human beings, expresses this more precisely than the linearly spaced frequency bands employed in the typical spectrum (Sharma et al., 2020).

The processes involved in obtaining MFCC characteristics from an audio source are as follows:

- Make a signal's Fourier transformation using a windowed extracted signal.
- Utilizing triangular overlapping windows, project the derived spectral powers onto the mel scale.
- Consider the list of mel log powers' discrete cosine transforms as a signal.

Equation 1 shows that the mathematical extraction for MFCCs is:


(1) $$\begin{array}{l} \text{ci}=\overset{N_{f}}{\underset{n=1}{\sum }}S_{n}\cos\left[i\left(n-0.5\right)\frac{\pi }{N_{f}}\right] \end{array}$$

where

- ci = cy(i) = ith MFCC coefficient.
- *N*_*f*_ Denotes triangular filters numbers in the filter bank.
- *S*_*n*_ is output of log energy outcomes *nth* filter coefficient.
- *L* shows MFCC coefficients, which we are interested in calculating.

#### 3.2.2 Frequency domain features

In signal processing and machine learning, frequency domain features are features taken from a signal's frequency domain representation. These properties are acquired using techniques such as the Fourier or wavelet transforms to convert the time-domain signal into the frequency domain. The spectrum or frequency domain representation sheds light on the signal's periodic components. Depending on the analytic technique and various frequency domains, information may be recovered, including wavelet coefficients, power spectral density, and reflection coefficients. These characteristics are widely utilized in audio signal processing and analysis applications. The frequency domain feature we used in our study is discussed below (Sharma et al., 2020).

##### 3.2.2.1 Spectral centroid

The spectral centroid (SC) is a measurement used to define a spectrum during digital data processing. It is calculated by averaging the frequencies in the signal and applying weights based on their magnitudes. The spectral centroid is commonly used in music information retrieval and is typically a trustworthy predictor of the “brightness” of a sound (Butt, 2012). The spectral centroid for a signal “y” may be obtained using Equation 2:


(2) $$\begin{array}{l} \text{centroid}\left[t\right]=\frac{\sum_{m}F\left[m,t\right]\cdot \text{freq}\left[m\right]}{\sum_{j}F\left[j,t\right]} \end{array}$$

where “F” is a magnitude spectrogram and “freq” is the array of frequencies (e.g., FFT frequencies in Hz) of the rows of “F.”

#### 3.2.3 Time domain features

Time domain features are properties obtained from a signal's time-based representation and are widely used in fields such as machine learning and signal processing (Butt, 2012). In this section, time domain features considered in this study such as RMS, STFT, and Mel-spectrogram are explained.

##### 3.2.3.1 RMS

The signal's root mean square (RMS) value is used to calculate the signal's magnitude. It is calculated by squaring the result after determining the square root of the average of the signal's squared values. The RMS value, which may be used to determine an audio source's power, is often employed when comparing the loudness of different audio sources.


(3) $$\begin{array}{l} x_{\text{RMS}}=\sqrt{\frac{1}{n}\overset{n}{\underset{i=1}{\sum }}x_{i}^{2}} \end{array}$$

In the above Equation 3, √ is used to represent the square root function, $\frac{1}{n}$ is used to represent division, $\overset{n}{\underset{i=1}{\sum }}$ is used to represent the summation from *i* = 1 to *n*, and $x_{i}^{2}$ represents the square of each value *x*_*i*_.

#### 3.2.4 Time-frequency domain features

Features in the time–frequency domain are obtained by jointly representing a signal in the frequency and time domains. These characteristics are often used when analyzing non-stationary signals whose frequency content fluctuates over time. Below is the time–frequency domain feature we are using in our study.

##### 3.2.4.1 Chroma STFT

Chroma features are an unusual and powerful representation of music audio, dividing the whole spectrum into 12 bins to reflect the 12 distinct chroma of the musical octave. Since notes that are exactly one octave apart are detected as being very similar in music, knowing how to distribute chroma without knowing its precise location (i.e., the original octave) may still provide significant musical details regarding the audio and thus yield a strong and compact representation.


(4) $$\begin{array}{l} \text{Chroma}\left[n,t\right]=\frac{1}{Z}\underset{m}{\sum }|X\left[m,t\right]|\cdot \text{chroma\_map}\left[m,n\right] \end{array}$$

In the above Equation 4:

- *n* represents the chroma bin (0 to 11).
- *t* represents the time frame.
- *X*[*m, t*] represents the complex spectrum at bin *m* and time *t*.
- chroma_map[*m, n*] is 1 if bin *m* belongs to chroma *n*, and 0 otherwise.
- *Z* is a normalization factor (for example, the sum of the magnitudes of all spectra).

##### 3.2.4.2 Mel-spectrogram

A mel-spectrogram is produced by translating the frequencies of a spectrogram to the mel scale. Listeners interpret the tones on the mel scale to be evenly spaced away from one another. The reference point between this scale and traditional measurement of frequency is produced by delivering a tone at 1,000 Hz that is 40 dB beyond the listener's threshold and has a perceived pitch of 1,000 mels. The listener perceives greater pauses to produce similar pitch increments over 500 Hz.


(5) $$\begin{array}{l} \text{MelSpec}\left[n,t\right]=\log\left(1+\frac{1}{N}\overset{N}{\underset{k=1}{\sum }}|X\left[k,t\right]{|}^{2}\cdot \text{mel\_filter}\left[n,k\right]\right). \end{array}$$

In the above Equation 5:

- *n* represents the Mel bin.
- *t* represents the time frame.
- *X*[*k, t*] represents the complex spectrum at bin *k* and time *t*.
- mel_filter[*n, k*] is the Mel filter response for Mel bin *n* at frequency bin *k*.
- *N* is the total number of frequency bins.
- log is the natural logarithm.

### 3.3 Machine learning models

Three machine learning models, namely logistic regression, linear discriminant analysis (LDA), and Random Forest Extra Trees, have been developed in this study to classify acoustic activities. These are the most frequent ML models applied in the different applications of acoustic activity classification in the various applications (Bansal and Garg, 2022).

#### 3.3.1 Extra Trees

Extra Trees is an ensemble strategy that boosts accuracy and stability by integrating the forecasts of several decision trees. More trees introduce more randomness into the tree-building process than past tree-based techniques, which can increase generalization and reduce over-fitting. Extra Trees, also known as Extremely Randomized Trees, is an ensemble learning algorithm similar to Random Forest. It combines multiple decision trees in a slightly different way.

#### 3.3.2 Linear discriminant analysis

Linear discriminant analysis (LDA) may reduce dimensionality by decreasing noise and processing complexity, thereby enhancing classification performance.

LDA equation is as follows. In Equation 6,


(6) $$\begin{array}{l} \frac{{\text{W}}^{T}\cdot \text{X}}{\sigma^{2}}=\frac{\mu_{1}-\mu_{2}}{\sigma^{2}}+\ln\left(\frac{P\left(\text{X}|\omega_{1}\right)}{P\left(\text{X}|\omega_{2}\right)}\right) \end{array}$$

where **W** is the weight vector, **X** is the input data vector, μ_1_, μ_2_ are class means, σ^2^ is the within-class variance, and *P*(**X**|ω_1_), *P*(**X**|ω_2_) are class-conditional probability densities.

#### 3.3.3 Logistic regression

Logistic regression is ideal for measuring predictability. The probabilistic technique determines the likelihood that input matches a given class. It provides readability, simplicity, robustness, and rapid learning utilizing likelihood estimates. The logistic regression equation is as follows in Equation 7:


(7) $$\begin{array}{l} P\left(Y=1|\text{X}\right)=\frac{1}{1+e^{-\left(\beta_{0}+\beta_{1}X_{1}+\beta_{2}X_{2}+\ldots +\beta_{n}X_{n}\right)}} \end{array}$$

The probability of *Y* being 1, given the input vector **X**, is modeled using logistic regression, with β_0_ to β_*n*_ as coefficients. The sigmoid function ($\frac{1}{1+e^{-z}}$) ensures the output lies between 0 and 1.

#### 3.3.4 Random forest

Random forest is a machine-learning algorithm that usually works well with high-dimensional problems and allows for non-linear interactions between predictors. However, the availability of linked predictors has been demonstrated to influence its capability to identify powerful predictors. The Random Forest-Recursive Feature Elimination approach (Random Forest RFE-SHAP) addresses this issue with limited data.

### 3.4 Deep learning models

We chose to employ RNN, LSTM, and RCNN in our work on classifying audio activities in the domestic environment. These deep learning methods are ideally suited for numerous acoustic classification challenges, including speech recognition, audio event detection, and acoustic scene classification.

#### 3.4.1 Recurrent neural network

Three layers comprise the recurrent neural network (RNN) model, built using the sequential API. The first layer, SimpleRNN, has 64 units and uses the activation function of ReLU. The second and third levels, dense layers, have 32 and 12 units each and use the ReLU and softmax activation functions. The model is built utilizing the optimizer known as Adam, accuracy for performance assessment, and sparse categorical cross-entropy for loss computation. Then, the model is trained using 20% of the training data as a validation set for 100 epochs with a batch size of 32. The model is then tested on a test set, and the results, including the test loss and accuracy, are printed.

The output of a Simple RNN unit at time *t* is calculated using Equations 8 and 9:


(8) $$\begin{array}{l} h_{t}=\tanh\left(W_{hh}\cdot h_{t-1}+W_{xh}\cdot x_{t}+b_{h}\right) \end{array}$$

where **W**_*hh*_ and **W**_*xh*_ are weight matrices, *h*_*t*−1_ is the previous hidden state, *x*_*t*_ is the input vector, and *b*_*h*_ is the bias.


(9) $$\begin{array}{l} y_{t}=W_{hy}\cdot h_{t}+b_{y} \end{array}$$

where **W**_*hy*_ is the weight matrix, *h*_*t*_ is the hidden state, and *b*_*y*_ is the bias.

#### 3.4.2 Long short-term memory

The long short-term memory (LSTM model) uses the sequential API, starting with a 64-unit LSTM layer. The next two dense layers, using the ReLU and softmax activation functions, respectively, have 32 and 12 units each. The model's construction uses the Adam optimizer, accuracy for performance evaluation, and sparse categorical cross-entropy. Then, it is trained over 100 iterations with a batch size of 32 using 20% of the training data as a validation set. The model is then put to the test on a test set, and the results are reported along with the test loss and accuracy. The LSTM unit at time *t* is calculated using the following Equations 10–14:

Forget gate: 1. Forget gate:


(10) $$\begin{array}{l} f_{t}=\sigma \left(W_{f}\cdot \left[h_{t-1},x_{t}\right]+b_{f}\right) \end{array}$$

The forget gate at time *t*, *f*_*t*_, is the sigmoid function applied to the weighted sum of the previous hidden state (*h*_*t*−1_), current input (*x*_*t*_), and bias (*b*_*f*_).

2. Input gate:


(11) $$\begin{array}{l} i_{t}=\sigma \left(W_{i}\cdot \left[h_{t-1},x_{t}\right]+b_{i}\right) \end{array}$$

The input gate at time *t*, *i*_*t*_, is the sigmoid function applied to the weighted sum of the previous hidden state (*h*_*t*−1_), current input (*x*_*t*_), and bias (*b*_*i*_).

3. Cell state:


(12) $$\begin{array}{l} {\tilde{C}}_{t}=\tanh\left(W_{C}\cdot \left[h_{t-1},x_{t}\right]+b_{C}\right) \end{array}$$

The cell state at time *t*, ${\tilde{C}}_{t}$, is the hyperbolic tangent of the weighted sum of the previous hidden state (*h*_*t*−1_), current input (*x*_*t*_), and bias (*b*_*C*_).

4. Output gate:


(13) $$\begin{array}{l} o_{t}=\sigma \left(W_{o}\cdot \left[h_{t-1},x_{t}\right]+b_{o}\right) \end{array}$$

The output gate at time *t*, *o*_*t*_, is the sigmoid function applied to the weighted sum of the previous hidden state (*h*_*t*−1_), current input (*x*_*t*_), and bias (*b*_*o*_).

5. Hidden state:


(14) $$\begin{array}{l} h_{t}=o_{t}\cdot \tanh\left({\tilde{C}}_{t}\right) \end{array}$$

The hidden state at time *t*, *h*_*t*_, is the element-wise product of the output gate (*o*_*t*_) and the hyperbolic tangent of the cell candidate (${\tilde{C}}_{t}$).

#### 3.4.3 Recurrent convolutional neural network

This model's input layer, reshape layer, bidirectional LSTM layer, two dense layers, and output layer. The input layer accepts a one-dimensional array of 159 features, and a two-dimensional array is then created using the reshape layer. A 64-unit bidirectional LSTM layer, which processes the input forward and backward, receives the data after it has been reshaped. As a result, the model can account for context for each time step in the past and the future. Following a GlobalMaxPooling1D layer and a dense layer with 32 units utilizing the ReLU activation function, the LSTM layer's output is transmitted through. Twelve units with a softmax activation function comprise the final output layer appropriate for multi-class classification problems. The model's construction uses the Adam optimizer, sparse categorical cross-entropy for loss computation, and accuracy for performance assessment. Then, it is trained using 20% of the training data as a validation set for 100 epochs with a batch size of 32. The model is then tested on a test set, and the results, including the test loss and accuracy, are printed.

### 3.5 Sound source localization

In this developed framework, we are dealing with a multi-path environment, in which sound can come from multiple microphones. An object (person) can add multiple paths of the same sound. One path is the direct path and the second is caused by the reflection of the object. In this case, the direction of the array plays a significant role in dealing with array signal processing. The traditional algorithms deal with the direction of arrival (DOA) based on maximum likelihood methods, subspace methods, and delay-and-sum and minimum variance distortionless response (MVDR) methods (Molaei et al., 2024). The machine learning-based method works well when the signal-to-noise ratio is low, but it's computationally very complex, At the same time the, sub-space method also works well and is computationally efficient. However, the most frequent techniques used due to the advantage of the orthogonality of subspace were ESPRIT and MUSIC. It is worth mentioning that ESPRIT-based approaches can have a maximum number of uncorrelated sources in terms of the size of sub-arrays (Hu et al., 2014). On the other side, the computational complexity in terms of quantitative comparison shows less computational complexity as compared to other localization algorithms, which ultimately minimize the amount of resources required to execute that algorithm (Molaei et al., 2024). Other localization methods like beam-forming (Priyanka, 2017), time of arrival (Xu et al., 2011), Time difference of arrival (Motie et al., 2024), multiple signal classification and machine learning approach (Ziauddin, 2024), ESPRIT offers high accuracy for DOA estimation in favorable conditions, particularly with closely spaced sources. Moreover, it works well with array data and leverages spatial correlation.

To identify sound sources and calculate signal parameters, we have applied a high-resolution subspace-based method called ESPRIT (Estimation of Signal Parameters using Rotational Invariance Techniques) (Cobos et al., 2017) The ESPRIT approach uses the rotational invariance property of the signal subspace to estimate the sound sources' DOA. This method is extremely useful when high-resolution localization is required, as in the fields of robotics and array processing. In ESPRIT, there are two main phases.

To calculate the signal subspace, ESPRIT calculates the eigen decomposition of a spatial covariance matrix obtained from several sensors. The eigenvectors that correspond to the largest eigenvalues make up the signal subspace, providing details on the sound sources DOA.

Estimating the signal parameters, ESPRIT solves a least-squares problem for calculating the signal parameters, such as frequencies and DOAs, using the rotational invariance property of the signal subspace. Compared to other high-resolution techniques like MUSIC (Multiple Signal Classification), ESPRIT has the advantage of not requiring a search across the whole spatial domain, reducing computing complexity and speeding up execution times.

## 4 Results

In this study, we have proposed an integrated framework for classifying activities, localizing acoustic events, and sending the emergency signal if an emergency activity is detected in the intelligent home environment. Multiple state-of-the-art machine learning and deep learning classifiers have been applied for the detection of activities, as well as the ESPRIT algorithm to localize acoustic events. The following performance measure metrics were considered to evaluate the significance of the results: Average Accuracy, Precision, Recall, F1-Sore, RMSE, and MSE. These are the most frequent metrics used in the literature to assess the significance of ML and DL models.

**Figure 6:**
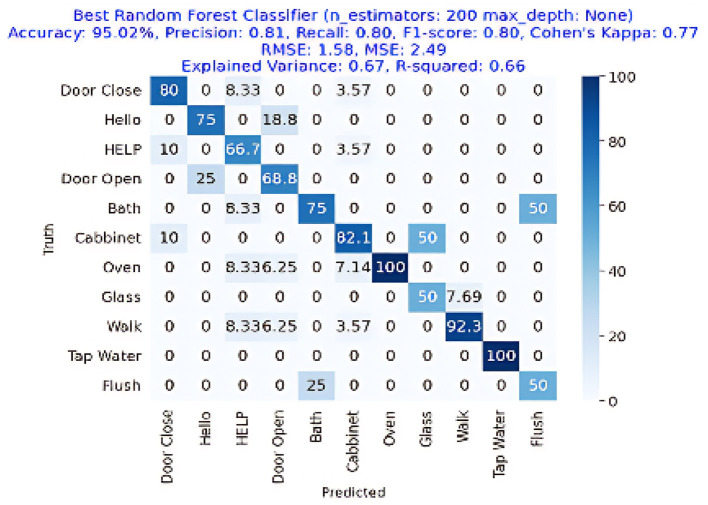
Confusion matrix for the best Random Forest Classifier, achieving an accuracy of 95.02%, precision of 0.81, recall of 0.80, and an F1-Score of 0.80.

**Figure 7:**
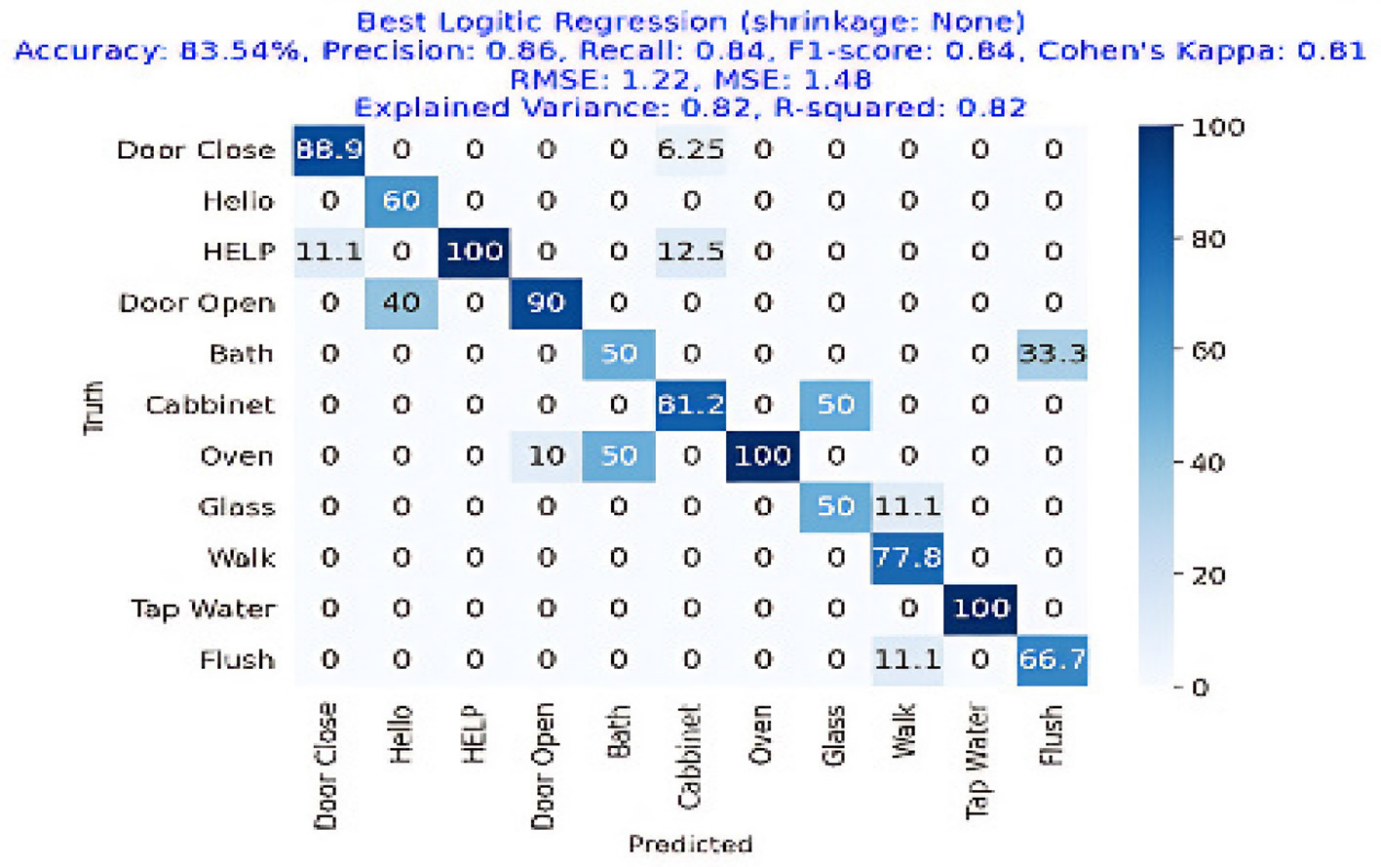
Confusion matrix for the best Logistic Regression classifier (no shrinkage), achieving an accuracy of 83.54%, precision of 0.86, recall of 0.84, and an F1-Score of 0.84.

**Figure 8:**
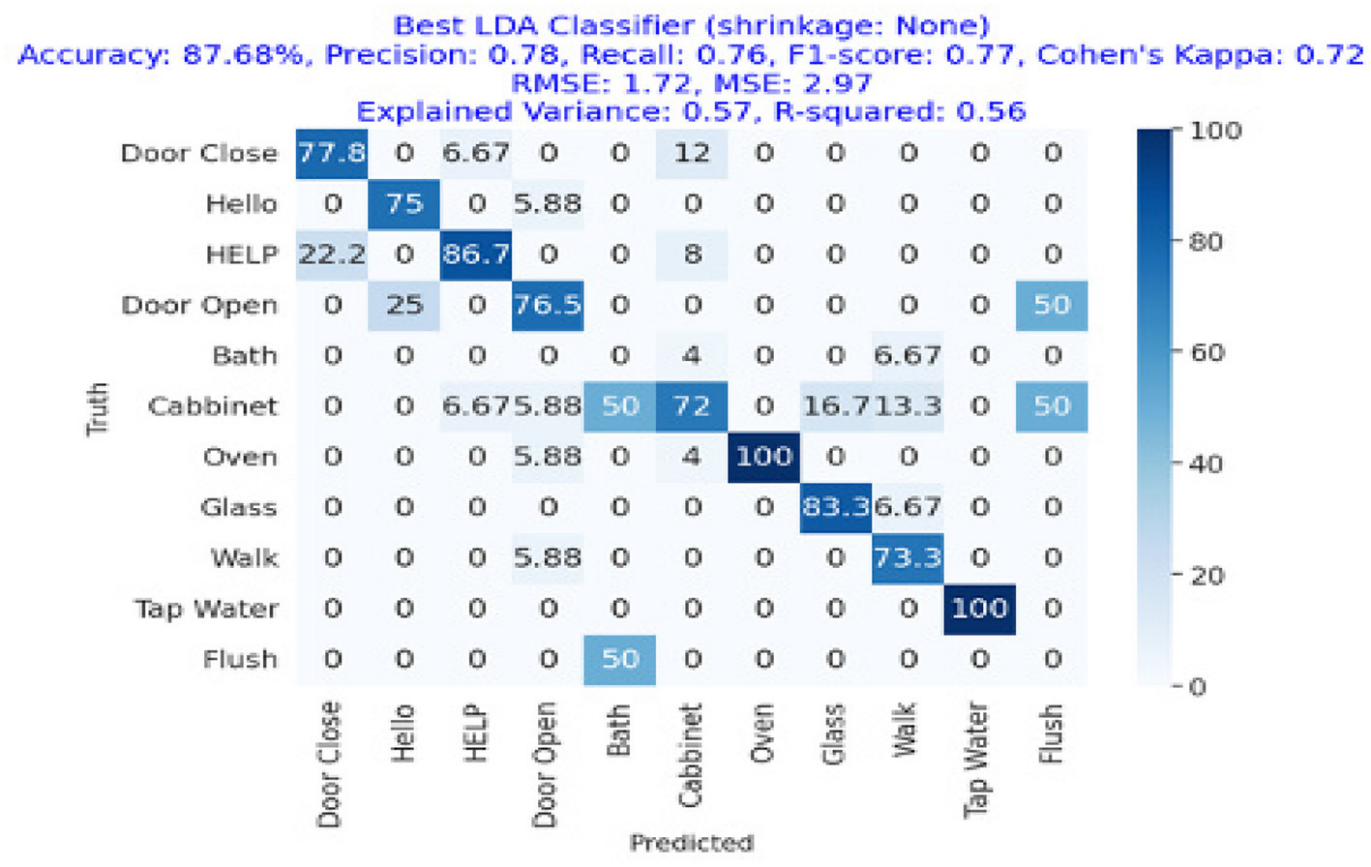
Confusion matrix for the best LDA classifier (no shrinkage), showing an accuracy of 87.68%, precision of 0.78, recall of 0.76, and an F1-Score of 0.77.

**Figure 9:**
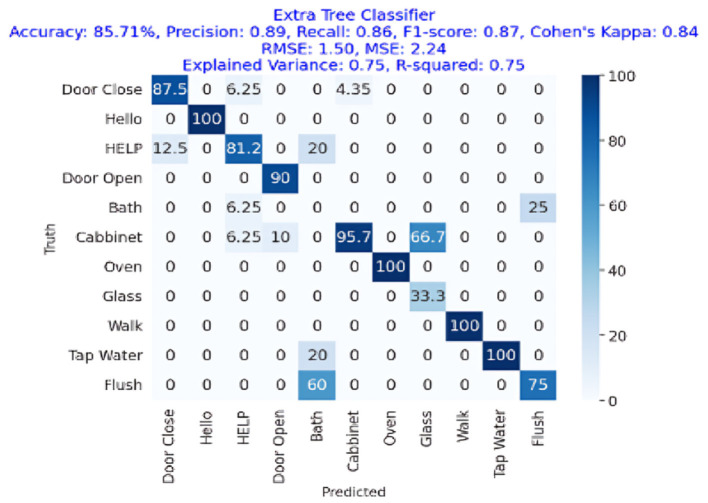
Confusion matrix for the Extra Tree Classifier in acoustic activity classification, achieving an accuracy of 85.71%, precision of 0.89, recall of 0.86, and F1-Score of 0.87.

The results are shown in Table 2. The classifier with the highest accuracy is the Random Forest (95.02%), as shown in Figure 6. This is followed by the LDA (87.68%) in Figure 7, the Extra Tree (85.71%) in Figure 8, and finally, logistic regression (83.54%), shown in Figure 9. This shows that the Random Forest classifier is the most successful out of all the evaluated models. However, the performance variations across the classifiers suggest that the classifiers selection and calibration for a given job may need some work.

**Table 2:** Results of activity detection using machine learning algorithms.

**ML classifier**	**Accuracy**	**Precision**	**Recall**	**F1-score**	**Rmse**	**Mse**
Random forest	95.02%	81%	80%	80%	1.58	2.49
LDA	87.68%	78%	76%	77%	1.72	2.97
LR	83.54%	86%	84%	84%	1.22	1.48
Extra tree	85.71%	89%	86%	87%	1.50	2.24

A more thorough activity-level classification analysis of the classifiers performance in various tasks is given in Table 3. Among the actions are doors closed, Hello, Help, Door Open, Bath, Cabinet, Oven, Glass Walk, Tap Water, and Flush. The classifier's performances differ for each task; some tasks are classified more correctly by certain classifiers than by others. For example, the best classifier for the “Hello” task was the Extra Tree classifier with a 100% detection, whereas the best classifier for the “Help” task was the Logistic regression classifier with a 100% detection. The detailed activity level results are presented in Table 3.

**Table 3:** Classification of activity levels using machine learning models.

**Activities**	**Random forest**	**LDA**	**Logistic regression**	**Extra tree**
Door close	80%	78%	88.9%	87.5%
Hello	75%	77%	60%	100%
Help	66%	86%	100%	81.2%
Door open	68.8%	76.5%	90%	90%
Bath	75%	50%	50%	20%
Cabinet	82%	72%	81%	96%
Oven	100%	100%	100%	100%
Glass walk	50%	33%	50%	67%
Tap water	92%	73%	78%	100%
Flush	100%	100%	100%	100%

The results of the deep-learning algorithms are shown in Table 4. The RCNN classifier, with an accuracy of 81.90%, is presented in **Figure 11**, outperforming the LSTM (81.01%) in **Figure 12** and the RNN (75.95%) shown in **Figure 13**. This implies that the LSTM classifier is the most successful out of all the evaluated models. However, the performance variations across the classifiers suggest that the classifier selection and calibration for a given job may need some work. A more detailed overview of the deep learning classifiers' performance in detection tasks is given in the Table 5. Among the actions are doors closed, Hello, Help, Door Open, Bath, Cabinet, Oven, Glass Walk, Tap Water, and Flush. The classifiers' performances differ for each task; some tasks are classified more correctly by certain classifiers than by others. For example, the “Door Close” task was most accurately classified by the RNN classifier, whereas the LSTM classifier most accurately classified the “Hello” task.

**Table 4:** Results of activity detection using deep learning models.

**DL classifier**	**Accuracy**	**Precision**	**Recall**	**F1-score**	**Rmse**	**Mse**
LSTM	81.01%	74%	81%	77%	1.46	2.14
RCNN	81.90%	83%	82%	82%	1.81	3.27
RNN	75.95%	70%	76%	72%	1.88	3.54
Residual DCNN	81.01%	74%	81%	77%	1.46	2.14

**Table 5:** Classification of activity levels using deep learning models.

**Activities**	**RNN**	**LSTM**	**RCNN**
Door close	78%	82%	80%
Hello	100%	100%	100%
Help	91%	91%	80%
Door open	78%	80%	89%
Bath	10%	30%	30%
Cabinet	70%	78%	91%
Oven	100%	100%	92%
Glass walk	40%	20%	50%
Tap water	60%	12%	93%
Flush	71%	71%	100%

Figure 6 represents the confusion matrix of Random Forest performance. The image depicts the 100% detection of Oven and Tap Water with more than 95% overall accuracy, showing the effectiveness of Random Forest classifier in the acoustic activities or event detection in the home environment.

Figure 7 represents the confusion matrix of logistic regression performance with more than 83% overall accuracy showing a great deal with logistic regression as getting an 80%+ accuracy in this task is hard. Figure 7 also depicts the superior performance of the classifier where logistic regression gained 86% precision, 84% recall, and 84% F1-Score and can be utilized in the real-time AAL applications.

Figure 8 represents the confusion matrix of linear discriminant analysis performance with more than 87% overall accuracy showing a great deal with linear discriminant analysis as getting an 80%+ accuracy as this task of ambient acoustic classification is hard. We can also see that the precision, recall, and F1-Score of the linear discriminant analysis approach were 78%, 76%, and 77%, respectively, while the accuracy was 87.68%.

Figure 9 presents the confusion matrix of Extra Tree Classifier performance showing that it has achieved more than 85% accuracy showing the effectiveness of acoustic events detection with the Extra Tree Classifier. The figure also shows that the Extra Tree Classifier algorithm's F1-Score, recall, and precision were 89%, 86%, and 87%, respectively. Cohen's Kappa score showed a high degree of agreement, which was 84%. The RMSE and MSE have respective values of 1.50 and 2.24. The explained variance and R square were both 0.75.

**Figure 10:**
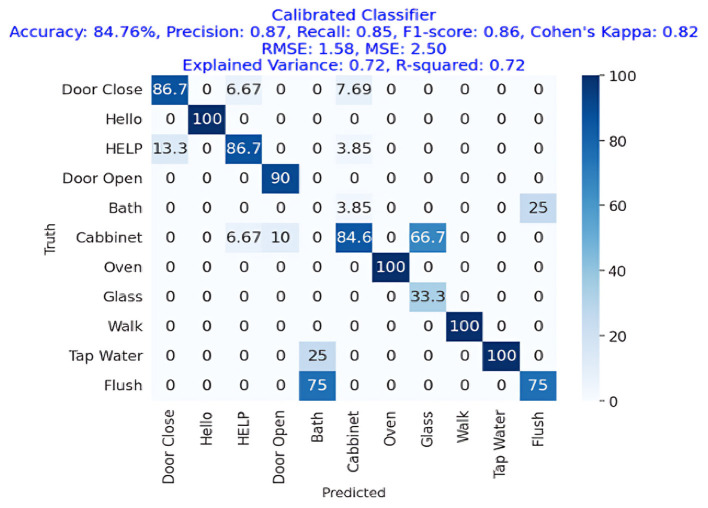
Confusion matrix of a calibrated classifier for acoustic activity classification, achieving an accuracy of 84.76%, precision of 0.87, recall of 0.85, and an F1-Score of 0.86.

Figure 10 shows the confusion and performance metrics, respectively. The figures showing the calibrated classifier approach achieved an accuracy of 84.76%, with comparable precision, recall, and F1-Score values of 87%, 85%, and 86%, respectively. The Cohen's Kappa score was 82%, which is a high level of agreement. The difference between the RMSE and the MSE was 1.58. The explained variance and R square were both 0.72.

### 4.1 Deep learning results

**Figure 11:**
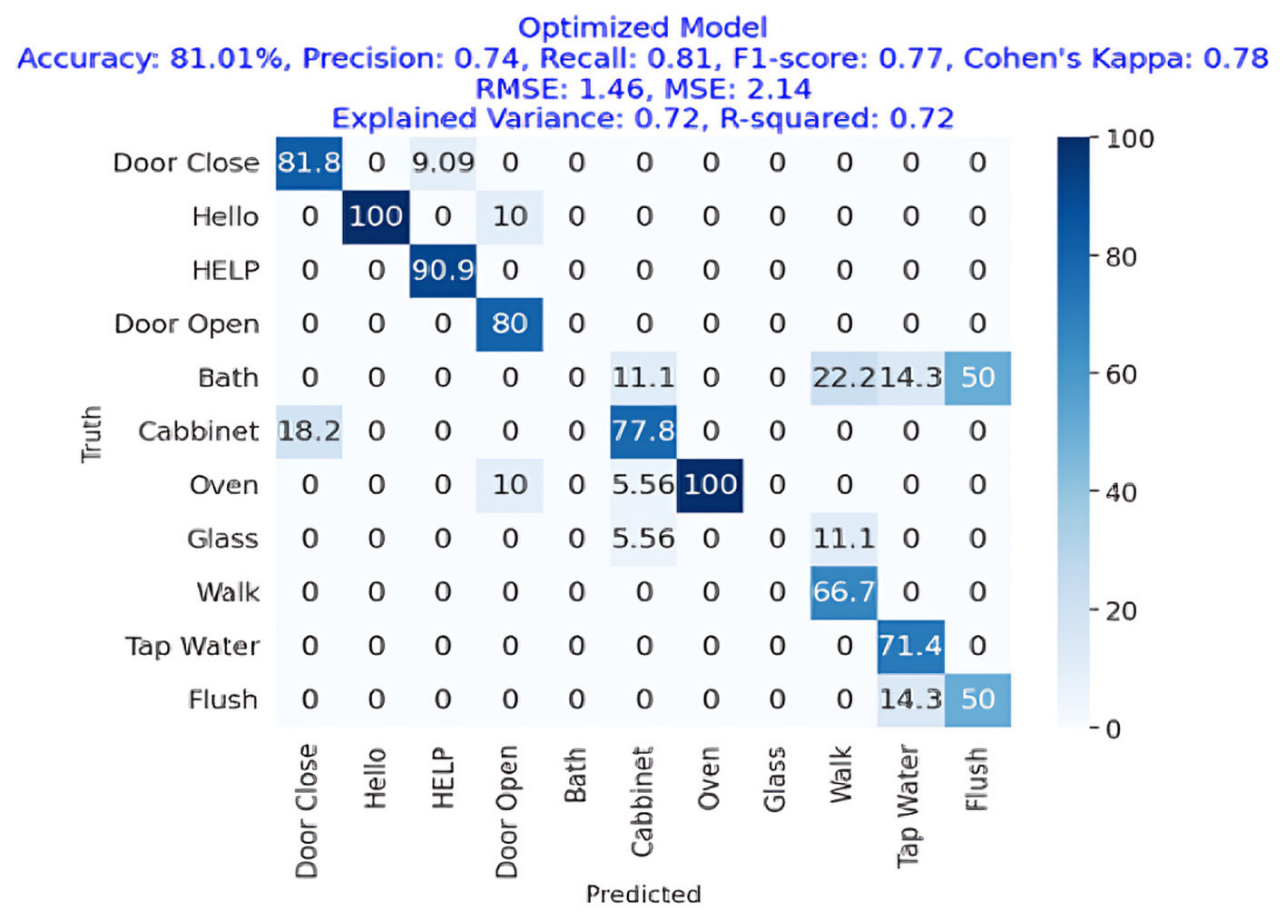
Confusion matrix of an optimized LSTM model for acoustic activity classification, showing an accuracy of 81.01% with strong recall 0.81 and F1-Score 0.77.

Figure 11 presents the confusion matrix of LSTM showing that LSTM has gained more than 81% accuracy, representing the strength of LSTM for AED in a home environment. It also shows the precision, recall, and F1-Score for the LSTM approach, which is employed in deep learning models, were each 74%, 81%, and 77%, respectively, for an accuracy of 81.01%. Cohen's Kappa score showed a high degree of agreement, which was 78%. The difference between the RMSE and the MSE was 1.46. R square and variance have 72% values each.

**Figure 12:**
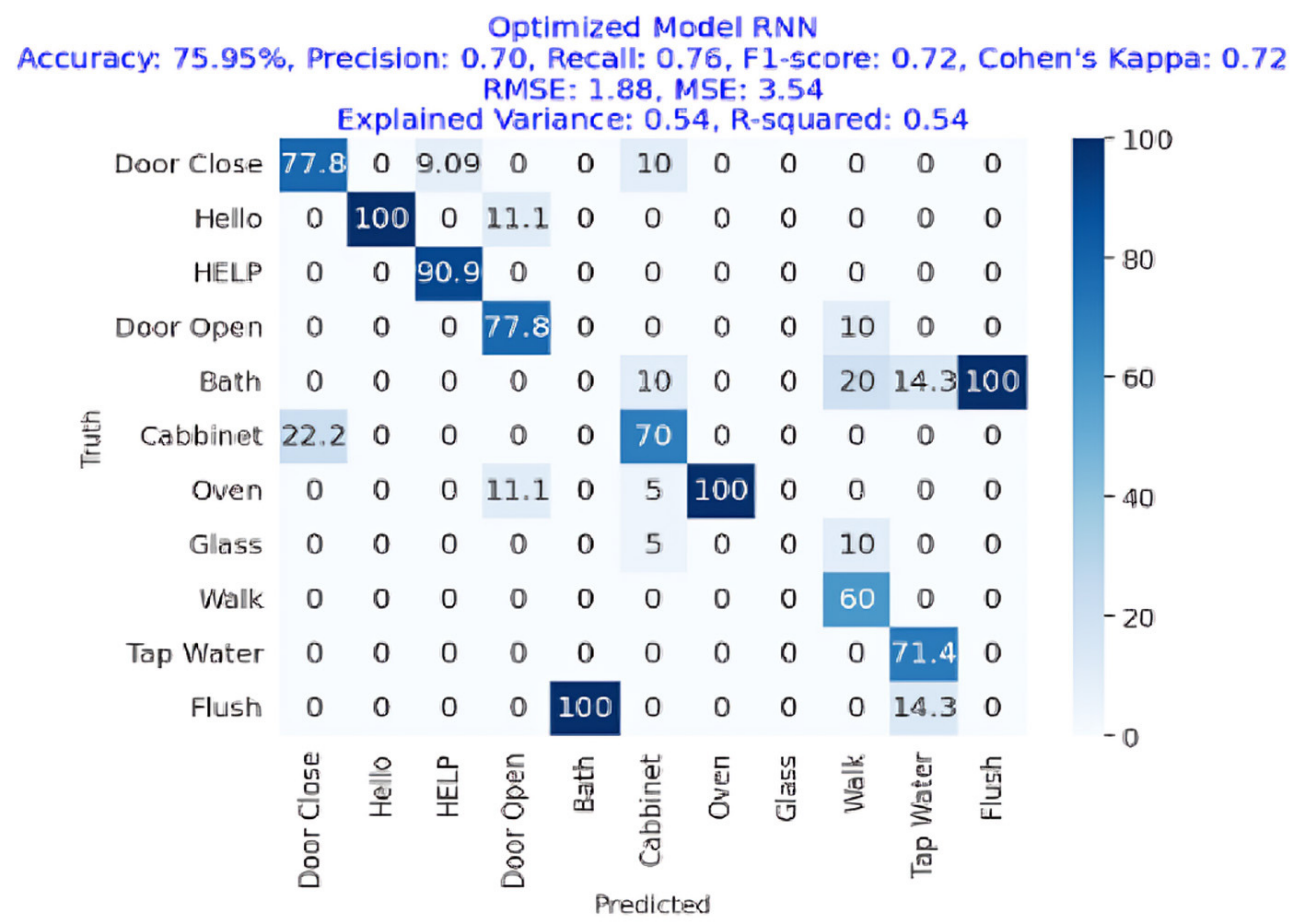
Confusion matrix of an optimized RNN model for acoustic activity classification, showing an accuracy of 75.95%, with a recall of 0.76 and an F1-Score of 0.72.

Figure 12 presents the confusion matrix of RNN showing that RNN has obtained more than 75% accuracy representing the performance of RNN for AED in a home environment. The RNN approach was accurate with an accuracy of 75.95%, precision of 70%, recall of 76%, and F1-Score of 72%. The Cohen's Kappa score was 72%, which is very congruent. The RMSE and MSE have respective values of 1.88 and 3.54. R square and variance explained both had values of 54%.

**Figure 13:**
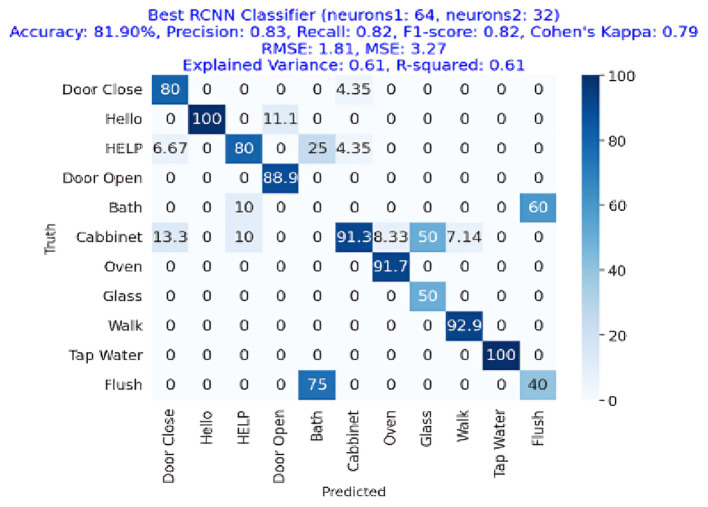
Confusion matrix for the best RCNN classifier achieving an accuracy of 81.90%, precision of 0.83, recall of 0.82, and F1-Score of 0.82.

**Figure 14:**
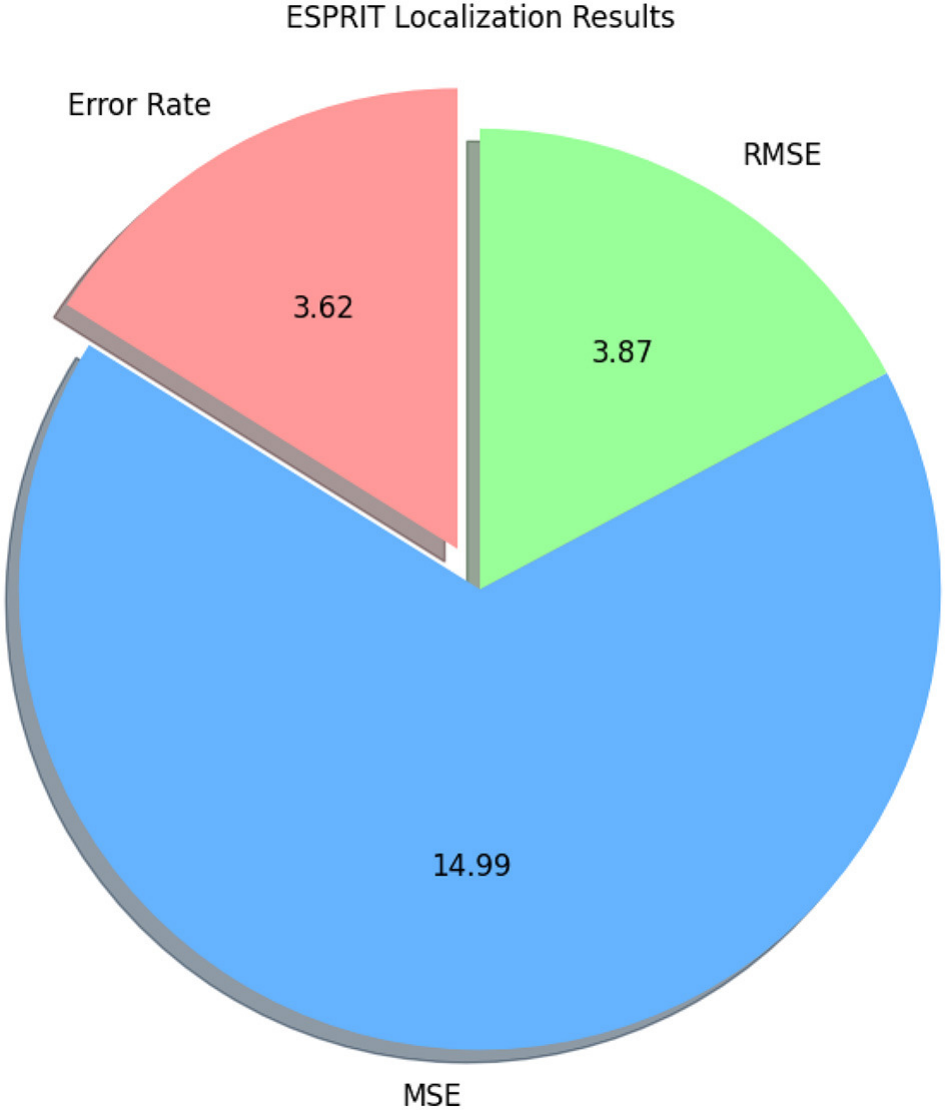
Pie chart illustrating ESPRIT localization results, showing an error rate of 3.62, Rmse of 3.87, and Mse of 14.99.

Figure 13 depicts the confusion matrix of RCNN, showing that RCNN has obtained more than 81% accuracy, representing the performance of the RCNN algorithm in the home Environment. The RCNN model's accuracy was 81.90%, with corresponding values of 82% precision, 83% recall, and 82% F1-Score. Cohen's Kappa score showed a high degree of agreement, which was 79%. The RMSE and MSE have respective values of 1.81 and 3.27. The explained variance and R square were both 61%. The study also used the ESPRIT algorithm for localizing the sources of activities. We obtained a comparatively low error rate of 3.62% shown in Table 6 and in Figure 14. It exhibited higher RMSE (3.87) and MSE (14.99) values due to the small data.

**Table 6:** Localization results using the ESPRIT algorithm.

**Metric**	**Value**
Error rate	3.62%
Mean squared error (Mse)	14.99
Root mean squared error (Rmse)	3.87

### 4.2 Emergency signal

**Figure 15:**
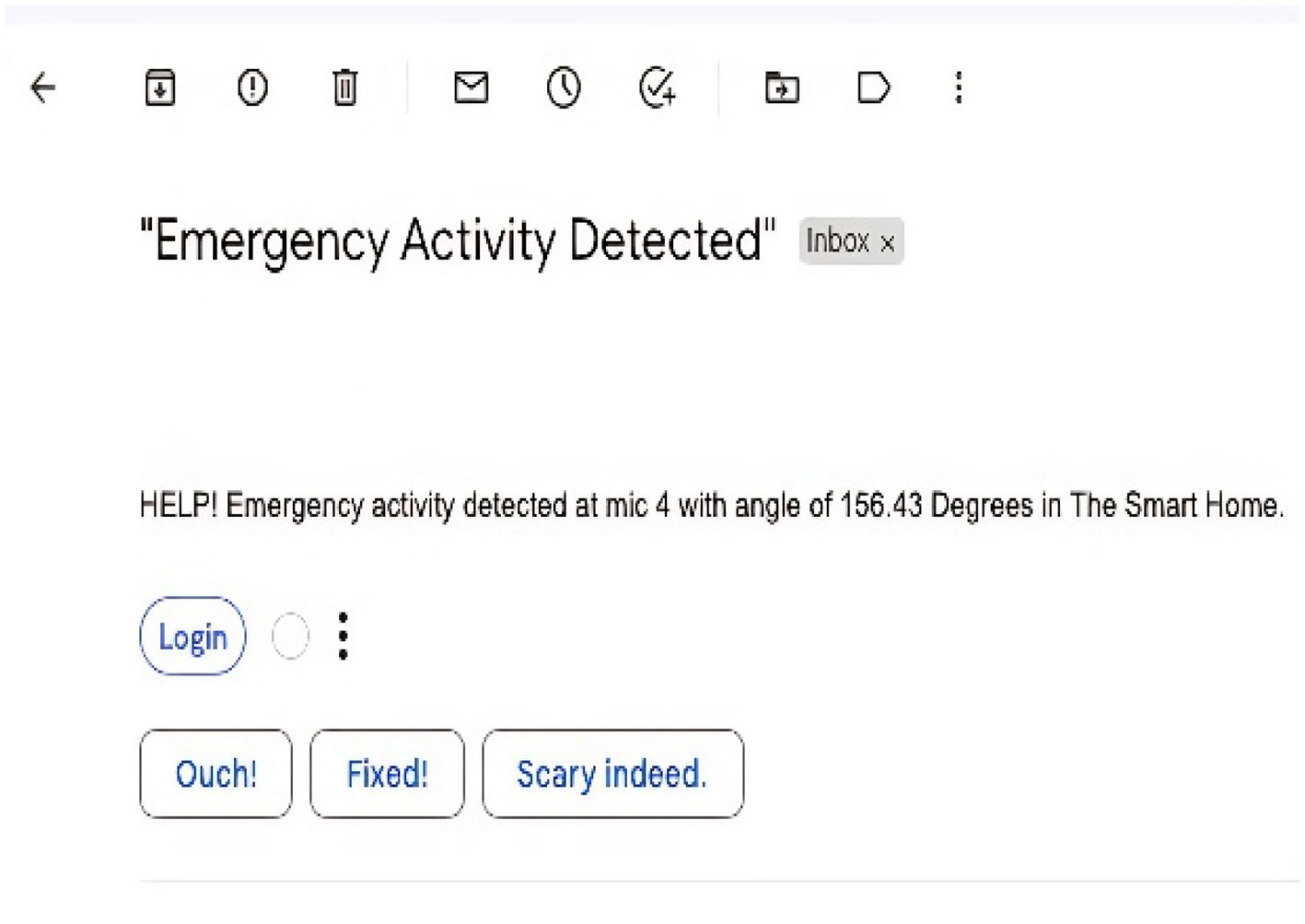
Emergency activity detection and emergency email sent.

Predicted class labels: [“HELP,” “Door close,” and “Door Open”] An emergency Email has been sent to ******@students.au.edu.pk. The above message was the output from our final framework when tested on a sample recording with four classes from our test data set. The activity we have trained our system to consider as an emergency activity, “HELP,” was also included in that sample data. The framework has correctly identified the activities, and on encountering “HELP,” it has successfully sent an emergency signal via electronic mail. The sent signal is presented in Figure 15 describing the emergency activity and the angles where it is detected in the Intelligent Home.

## 5 Comparison with state-of-the-art methods

Different experiment environments, data, and evaluation methods are applied to classify different numbers of events in the home environment in previous literature. Hence, it is quite difficult to make a direct comparison between different algorithms and methods in the existing literature due to differences in the framework of the home environment. However, we have attempted to compare our study with the methods described in the most recent five studies closely aligned with our framework, as described in Table 7 (Pandya and Ghayvat, 2021; Mondal and Barman, 2022; Giannoulis et al., 2019; Chin et al., 2021; Wu et al., 2023; Sasou et al., 2018). Most of the studies focused on one task at a time classification of the acoustic events or localization, and had complex architecture, which needed a lot of resources as well as a dedicated setup.

**Table 7:** Comprehensive comparison of SED approaches in recent and earlier studies.

**References**	**Events classified**	**Model**	**Accuracy**	**Localization**	**Complexity**	**Noise robustness**	**Validation**
Wu et al., 2023	Footstep ID/ Localization	CRNN/SVM	83.01% (CRNN)	Yes	High	Moderate	No
Mondal and Barman, 2022	11	GTDNN	88.5% (94.7% real-time)	No	High	Moderate	Yes
Chin et al., 2021	4	NN	74.6%	No	Low	Limited	No
Pandya and Ghayvat, 2021	22	LSTM-CNN	76.9%	No	High	Moderate	No
Giannoulis et al., 2019	Room SAD	NN	87.7%	Yes	Moderate	Limited	No

**1. Foundational machine learning and initial advancements**

Early SED research focused on machine learning models with limited complexity and adaptability. For instance, Sasou et al. (2018) aimed at improving sound event detection through noise reduction but lacked detailed classification metrics and comprehensive validations. These early models were effective for simple scenarios but were not scalable to real-world environments.

In contrast, our study used a comprehensive approach by using multiple machine learning classifiers, including Random Forest, which achieved an average accuracy of 95.02%. This surpasses the initial models that focused only on localization without a robust classification framework. Our use of multiple models provided better adaptability and comprehensive performance evaluations.

**2. Neural network applications and early deep learning approaches**

Giannoulis et al. (2019) emphasized sound detection with a neural network achieving an 87.7% accuracy. This was a significant milestone that demonstrated the potential of neural networks for SED. Similarly, Chin et al. (2021) used a context-aware framework for specific event detection, achieving a 74.6% accuracy. While these studies proved the efficacy of neural networks, their performance was limited by event diversity and comprehensive validations.

Our findings position the Random Forest classifier as a superior performer in machine learning-based SED, while the RCNN emerged as the best deep learning model with an average accuracy of 81.90%. Compared to the 87.7% achieved by Giannoulis et al. (2019), our model did not reach higher percentages in pure accuracy but excelled in terms of Precision, Recall, and F1-Score, highlighting its reliability in detecting true positive cases, which is crucial for emergency scenarios.

**3. Hybrid architectures and state-of-the-art benchmarks**

In 2021, Pandya and Ghayvat (2021) used LSTM–CNN hybrids, achieving a 76.9% accuracy on benchmark datasets. Their work set new standards by demonstrating that hybrid models could enhance detection capabilities and our study has attained 81% accuracy with the simple LSTM due to noise removal from signal and picking the right feature using recursive feature elimination technique Similarly, Mondal and Barman (2022) presented the GTDNN algorithm with an 88.5% accuracy for detecting 11 types of events, showcasing practical application potential with a 94.7% real-time detection rate. Mondal and Barman (2022) attained higher accuracy than our SED system, but the result was not validated with other metrics, showing the limitation of their study in understanding the model performance in real world environment. This indicates our model's potential for generalizing on unseen data with reliable accuracy, which is vital for real-world applications.

**4. Advancements with CRNN models and specific applications**

Wu et al. (2023) demonstrated the strength of CRNN models, achieving 83.01% accuracy for event detection, specifically in robotic applications. This approach underscored the versatility and adaptability of CRNNs for specialized tasks. In comparison, our RCNN, achieving an 81.90% accuracy with a focus on emergency detection and localization underscores its practical application, especially when coupled with our superior Random Forest classifier for broader classifications.

## 6 Discussion

Our study, which focused on using acoustic signals for activity classification and localization, offers significant advancements over earlier approaches. Unlike conventional sensor technologies that rely on visual data or direct user involvement, our acoustic-based framework offers a passive solution that respects privacy, requires minimal infrastructure, and can cover large areas. This advantage sets our approach apart from studies such as Pandya and Ghayvat (2021) shown in Table 7, which used visual sensing to classify activities, thereby introducing potential privacy concerns and higher setup costs. Our approach, in contrast, mitigates these challenges by leveraging sound as the primary data source, which not only reduces privacy risks but also lowers the cost of system deployment.

Additionally, compared to studies like Chin et al. (2021) and Wu et al. (2023) as mentioned in Table 7, which faced limitations in the number of activities covered and achieved lower F1-Scores, our methodology improves upon these aspects. Specifically, our system can detect and classify a wider range of activities with high accuracy. The Random Forest classifier, which achieved a remarkable 95.02% average accuracy, performs better than the classifiers in these studies, which were restricted by fewer activities and smaller datasets. Our results show that our framework delivers more reliable performance, both in terms of accuracy and robustness, when compared to earlier work.

However, as with many of the studies highlighted in Table 8, our framework faces challenges, particularly in dealing with environmental noise and the scalability of the system across diverse real-world scenarios. Studies like Giannoulis et al. (2019) and Sasou et al. (2018) discussed the limitations posed by room acoustics and ambient noise, which can degrade model accuracy. While our approach incorporates techniques like Wiener filtering and data augmentation to mitigate noise, these methods are not always sufficient in highly variable environments. In this regard, integrating multimodal data sources, such as combining audio with motion or temperature sensors, could help improve reliability and reduce the impact of noise, as suggested in Mondal and Barman (2022) and Yang et al. (2022). The detailed results of these research works are presented in Table 8. This would allow our system to adapt more effectively to noisy or uncontrolled environments, enhancing its overall performance and scalability.

**Table 8:** Feasibility of existing solutions to enhance our framework.

**References**	**Proposed solution**	**Key findings & challenges**
Mondal and Barman, 2022	Multimodal Sensing (Audio + Motion)	**Advantages:** Improved accuracy in noisy environments. **Challenges:** Data fusion complexity, high cost, more resources needed.
		**Challenges:** High cost, complex fusion, resource demands.
Yang et al., 2022	Multimodal Sensing (Audio + Motion, etc.)	**Advantages:** Robust detection with multiple sensors. **Challenges:** Real-time processing overhead from synchronization.
		**Challenges:** High real-time processing needs.
Mohaimenuzzaman et al., 2023	Edge Computing (Local data processing)	**Advantages:** Reduced latency, enhanced privacy. **Challenges:** Limited computational power for real-time recognition.
		**Challenges:** Resource constraints, struggles with real-time tasks.
Wu et al., 2023	Edge Computing (Optimized models)	**Advantages:** Reduced latency, efficient local processing. **Challenges:** Need for model optimization, trade-offs in complexity.
		**Challenges:** Model complexity compromises efficiency.
Thottempudi et al., 2024	Scalability	**Advantages:** Identifies challenges in real-world environments. **Challenges:** Poor performance in noisy, large environments.
		**Challenges:** Performance drop in noisy environments.
Chin et al., 2021	Scalability (Cross-environment evaluation)	**Advantages:** Importance of diverse datasets. **Challenges:** Adapting models from controlled to real-world Environments.
		**Challenges:** Limited generalization across environments.
Devagiri et al., 2024	Scalability (Domain adaptation)	**Advantages:** Continual learning for environmental adaptation. **Challenges:** Experimental, real-world domain adaptation challenges.
		**Challenges:** Domain adaptation still experimental.

Moreover, the localization component of our framework, utilizing the ESPRIT algorithm, performs exceptionally well with a localization error rate of just 3.62%. This is particularly advantageous when compared to studies like Mohaimenuzzaman et al. (2023), as its results demonstrate in Table 8. Their study explored edge computing for local data processing but faced difficulties due to limited computational power for real-time recognition. The ESPRIT algorithm's low error rate demonstrates its potential for real-world applications, allowing for both accurate activity classification and precise localization without the heavy computational demands often associated with deep learning-based systems.

In terms of scalability, challenges similar to those identified in Chin et al. (2021) and Thottempudi et al. (2024) persist, particularly when adapting models to real-world conditions as shown in Table 8. These studies noted that models often struggle to generalize across diverse environments, especially in noisy settings. Although our framework has made significant progress in managing these challenges, there is still room for improvement. We anticipate that incorporating more advanced data pre-processing techniques, such as noise suppression and the use of larger, more varied datasets, could improve the robustness and scalability of our system. Additionally, continuing to explore methods for domain adaptation, as discussed in Devagiri et al. (2024) and its findings are added in Table 8, could further help in ensuring that our system performs well in a range of real-world scenarios.

In conclusion, while our approach provides a strong foundation for activity detection and localization in acoustic settings, it also shares common limitations with prior research, such as sensitivity to environmental noise and challenges with real-time processing. The studies discussed in Table 8 offer valuable insights that can guide our framework's future development, particularly in improving robustness, scalability, and multi-modal integration. By addressing these challenges, we can further enhance the effectiveness and applicability of the system in real-world smart home environments, particularly for emergency detection and alerting applications. In the next sections, certain solutions are discussed that can enhance the efficiency of our proposed system when incorporated into our framework, further improving the developed model.

### 6.1 Multi-modal sensing for activities classification

Multi-modal sensing, which integrates acoustic data with other sensor types (e.g., motion, temperature, pressure), offers the potential to dramatically enhance activity detection accuracy, particularly in loud surroundings. As demonstrated by Mondal and Barman (2022), shown in Table 8, combining audio with motion sensors gives a more robust solution for human activity recognition, especially when faced with environmental noise. However, combining several sensors brings a number of challenges:

- **Data fusion:** Combining data from different sensors requires complex data fusion techniques to synchronize and blend the information properly.
- **Increased cost and complexity:** Multi-modal sensing systems tend to be more expensive and require additional computer resources for processing, perhaps making them less practical for large-scale implementation in households.
- **Real-time processing:** As stated by Yang et al. (2022) shown in the Table 8, the necessity to interpret input from many sensors in real-time adds computing overhead, which might influence the performance of activity recognition systems.

Overall, while multi-modal sensing has the potential to improve robustness, its implementation comes with higher system complexity, cost, and resource needs. These limitations must be carefully considered when evaluating multi-modal techniques for real-world implementation in smart homes.

### 6.2 Edge computing in smart home systems

Edge computing has been offered as a potential option to enhance the privacy, efficiency, and scalability of activity recognition systems. By processing data locally on edge devices rather than transmitting it to the cloud, edge computing reduces latency and assures that sensitive data is not transferred over the Internet, thus preserving user privacy. However, the possibility of incorporating edge computing in smart home systems for acoustic activities classification remains an unsolved problem, especially considering the following challenges:

- **Limited computational resources:** Edge devices often have limited processing power and memory, making it challenging to run complex deep learning models that are often necessary for high-accuracy activity recognition. As observed by Mohaimenuzzaman et al. (2023), mentioned in Table 8, edge devices may struggle with the computing needs of real-time, high-accuracy auditory recognition.
- **Model optimization:** Studies such as Wu et al. (2023), shown in Table 8, indicate that while edge computing can reduce latency, achieving sufficient processing power on edge devices requires significant optimization of the models utilized. This may include decreasing the model size or compromising on the complexity of the algorithms, which could influence overall system performance.
- **Scalability and deployment:** Deploying edge computing solutions at scale in varied household environments pose problems relating to hardware heterogeneity, device compatibility, and network infrastructure. Mohaimenuzzaman et al. (2023), as mentioned in Table 8, claim that edge computing could increase performance in controlled situations, its scalability across diverse houses and smart devices needs further research.

While edge computing offers privacy and efficiency benefits, further research is needed to optimize models for resource-constrained devices and test their real-world scalability. The processing limits of edge devices remain a significant problem in smart home activity identification.

### 6.3 Scalability and generalization of activity recognition models

Scalability and generalization of activity recognition models are another significant difficulty. While the model performs well in controlled situations, real-world scenarios include diversity in room layouts, noise levels, and user behaviors. This issue is explored extensively in the literature, where studies like Thottempudi et al. (2024) and Chin et al. (2021) shown in Table 8 indicate that models trained in one environment often fail to transfer effectively to others. Our study similarly showed a performance decrease when the model was evaluated in situations that differed from the training data.

- **Diverse environments:** The performance of the model was dramatically lowered in larger rooms or locations with higher background noise. This shows that training on more diverse datasets, which depict a wide range of real-world situations, will be important to increase generalization.
- **Adaptation to new environments:** Continual learning and domain adaptation strategies, as mentioned by Devagiri et al. (2024), shown in Table 8, could assist in addressing these problems by allowing the model to adjust to changing surroundings over time.

Improving the generalization capabilities of activity recognition models will require a combination of more diversified datasets and sophisticated strategies for domain adaptation.

## 7 Future work

Although our analysis of acoustic event detection and localization in smart home shows promising results, there are still several limitations of this study. First, our study is based on a single smart home data with one person in the house. Due to the limited availability of data, we were not able to test the framework on multiple homes or dynamic environments. The limitation can be fulfilled upon the availability of the data.

The second limitation is the use of the conventional algorithm ESPRIT for sound localization. Although it has produced better results than expected, there are still certain limits that open up new avenues for future research. The main obstacle is the use of traditional microphone arrays for sound detection, which are effective but have scalability and flexibility issues. We suggest investigating sophisticated neural network models trained with information from sound sensors positioned in key areas to get over these obstacles and maybe boost the effectiveness of sound localization. Without the need for large microphone arrays, such models may be able to understand intricate sound patterns and the acoustics of their surroundings, resulting in more precise and effective sound localization.

To validate the developed models and enhance their generalization ability, the authors can utilize publicly available datasets that comprise a broad range of acoustic events and environments. By selecting diverse datasets such as the UrbanSound dataset or ESC-50, the authors can conduct cross-dataset evaluations, training their models on one dataset and testing them on another to assess robustness and performance. Implementing domain adaptation techniques, such as fine-tuning and transfer learning, will further enable the models to modify to the unique characteristics of distinct datasets. Comprehensive performance metrics–such as accuracy, precision, and F1-Score–should be employed to provide a comprehensive evaluation of the models. Additionally, comparing the models' performance against state-of-the-art approaches will help emphasize their strengths and areas for refinement. This validation process will not only reinforce the models' efficacy across varied acoustic contexts but also identify limitations that need to be addressed in future work, ensuring a continuous feedback cycle for model enhancement.

In addition to the challenges in sound localization, the discipline of acoustic event detection could significantly benefit from adaptable learning techniques. By integrating reinforcement learning into the acoustic event detection framework, models can dynamically adjust to variations in sound characteristics and contexts. This adaptability allows for real-time refinement based on feedback from misclassifications, considerably augmenting the model's ability to distinguish between comparable acoustic events effectively. Employing such techniques not only addresses the issue of misclassification across different activities but also reduces false predictions, thereby increasing overall classification accuracy. Furthermore, the utilization of publicly available datasets in combination with adaptable learning strategies will provide opportunities for the models to generalize better across diverse environments, ultimately leading to more robust acoustic event detection systems.

Additionally, new directions for study in acoustic event detection and localization within smart home environments are opened up by the integration of location estimation sensor technologies. This method promises to improve localization systems' accuracy and efficiency while also being scalable and adaptable to a variety of situations. Future research is expected to explore these possibilities using machine learning's advantages to overcome the shortcomings of existing approaches and advance scientific understanding in this field. Moreover, there is some confusion between different events during classification. Since there is overlap between different event gestures, it may lead to false-positives and-negatives. In future work, the focus should be on de-noising techniques, as proposed in recent studies (Othman et al., 2022; Iqbal, 2023), which can help overcome the misclassification of different events.

## 8 Conclusion

The study's findings provide insightful information on how different deep learning and machine learning classifiers perform when identifying and localizing acoustic activity. Random Forest, LDA, logistic regression, Extra Tree, LSTM, RCNN, and RNN are the classifiers developed for activity detection and classification from acoustic sound as well as the assessment of performance of the ESPRIT algorithm for localization. Out of all the machine learning classifiers, the Random Forest classifier has achieved the highest accuracy with an average accuracy of 95.02% for the acoustic activity classification. However, the other classifier, logistic regression, had the highest recall and average precision of 86% and 84% respectively, indicating its increased dependability in detecting true positive cases and minimizing false negatives with logistic regression. Conversely, the RCNN classifier has the maximum average accuracy (81.90%) as well as the greatest rates of precision, recall, and F1-Score among all deep learning classifiers. Lower RMSE and MSE values for the LSTM classifier suggest the model has well detected the pattern of different acoustic activities, suggesting that the model has a good generalizing ability on unseen data. Additionally, the ESPRIT algorithm yielded encouraging results, with an error rate of 3.62%. The lower error rate for localization with ESPRIT suggests the algorithm's superior performance and its implications in real-world applications for joint acoustic activity detection and localization. Similarly, it presents the emergency event detected by the Random Forest algorithm and activity located by the ESPRIT Algorithm when we tested the system on the help signal taken as a testing sample from our dataset.

In conclusion, our study has shown the superiority of Random Forest as the best machine learning classifier, RCNN as the best deep learning classifier, and the ESPRIT technique for acoustic source localization. By configuring these parameters, an emergency alert system in the real world can detect and locate sound activity while also transmitting emergency signals. The study also discovered that our approach to work should be dictated by its particular needs. It also emphasized the necessity for more studies to discover new approaches and enhance current ones, particularly in raising the precision of deep learning models and localization outcomes using large data sets. These discoveries are crucial for developing more advanced and effective systems to locate and detect sound activity, particularly for promptly delivering emergency warnings.

## Author's note

The authors declare the transparency of this study. To facilitate replication, the authors commit to providing a comprehensive tutorial for reproducing the study. Additionally, the dataset and experimental scripts will be made available upon request and open-sourced to support further research and verification.

## Data Availability

The raw data supporting the conclusions of this article will be made available by the authors, without undue reservation.
